# Inner tegument proteins of Herpes Simplex Virus are sufficient for intracellular capsid motility in neurons but not for axonal targeting

**DOI:** 10.1371/journal.ppat.1006813

**Published:** 2017-12-28

**Authors:** Anna Buch, Oliver Müller, Lyudmila Ivanova, Katinka Döhner, Dagmara Bialy, Jens B. Bosse, Anja Pohlmann, Anne Binz, Maike Hegemann, Claus-Henning Nagel, Martin Koltzenburg, Abel Viejo-Borbolla, Bodo Rosenhahn, Rudolf Bauerfeind, Beate Sodeik

**Affiliations:** 1 Institute of Virology, Hannover Medical School, Hannover, Germany; 2 NRENNT–Niedersachsen Research Network on Neuroinfectiology, Hannover, Germany; 3 DZIF—German Center for Infection Research, Hannover, Germany; 4 Institute for Information Processing, Leibniz University, Hannover, Germany; 5 REBIRTH—From Regenerative Biology to Reconstructive Therapy, Hannover, Germany; 6 Heinrich-Pette-Institute, Leibniz-Institute for Experimental Virology, Hamburg, Germany; 7 Institute of Neurology, University College, London, United Kingdom; 8 Research Core Unit Laser Microscopy, Hannover Medical School, Hannover, Germany; University of Wisconsin-Madison, UNITED STATES

## Abstract

Upon reactivation from latency and during lytic infections in neurons, alphaherpesviruses assemble cytosolic capsids, capsids associated with enveloping membranes, and transport vesicles harboring fully enveloped capsids. It is debated whether capsid envelopment of herpes simplex virus (HSV) is completed in the soma prior to axonal targeting or later, and whether the mechanisms are the same in neurons derived from embryos or from adult hosts. We used HSV mutants impaired in capsid envelopment to test whether the inner tegument proteins pUL36 or pUL37 necessary for microtubule-mediated capsid transport were sufficient for axonal capsid targeting in neurons derived from the dorsal root ganglia of adult mice. Such neurons were infected with HSV1-ΔUL20 whose capsids recruited pUL36 and pUL37, with HSV1-ΔUL37 whose capsids associate only with pUL36, or with HSV1-ΔUL36 that assembles capsids lacking both proteins. While capsids of HSV1-ΔUL20 were actively transported along microtubules in epithelial cells and in the somata of neurons, those of HSV1-ΔUL36 and -ΔUL37 could only diffuse in the cytoplasm. Employing a novel image analysis algorithm to quantify capsid targeting to axons, we show that only a few capsids of HSV1-ΔUL20 entered axons, while vesicles transporting gD utilized axonal transport efficiently and independently of pUL36, pUL37, or pUL20. Our data indicate that capsid motility in the somata of neurons mediated by pUL36 and pUL37 does not suffice for targeting capsids to axons, and suggest that capsid envelopment needs to be completed in the soma prior to targeting of herpes simplex virus to the axons, and to spreading from neurons to neighboring cells.

## Author summary

Human and animal alphaherpesviruses establish lifelong latent infections in neurons of the peripheral nervous system and cause many diseases upon primary infection as well as following reactivation from latency. The highly prevalent human herpes simplex viruses HSV-1 and HSV-2 are responsible for facial and genital herpes, potentially blinding eye infections, and life-threatening encephalitis and meningitis. Here, we asked how these viruses master the bottleneck of being targeted from the neuronal somata to the axons. Our data suggest that only transport vesicles harboring fully matured virus particles can enter axons for spreading infection to the brain or the peripheral organs. Our data imply that the limiting membrane of the transport vesicles must expose viral or host receptors to recruit the microtubule motors required for axonal transport. Inhibiting such viral factors on the surface of the transport vesicles might provide novel therapeutic approaches to prevent the spread of alphaherpesviruses in the nervous system.

## Introduction

Many pathogens spread within the nervous system by fast intracellular axonal transport mediated by microtubule motors, and progeny particles are transmitted via synapses from neuron to neuron [1,2]. Such life style allows pathogens to hide from the extracellular complement system, from antibodies, and from immune cells. But it comes with the challenge of finding the tiny exit gates from the neuronal somata, and of specific targeting to dendrites or to axons. It has been estimated that the cross section of the axonal outlet occupies less than 2 thousandth of the surface area of the soma [3,4]. The molecular post codes achieving this remarkable intracellular trafficking control are not well understood; neither for host nor for pathogen cargoes.

Alphaherpesviruses such as herpes simplex viruses (HSV) replicate in the skin, the eyes, and the oral, genital, or nasal mucosa, and then enter local axon endings of sensory or autonomic neurons in which they establish latency with little gene expression. Latent HSV-1 genomes have been detected in human cranial ganglia, especially in the trigeminal ganglia, while dorsal root ganglia (DRG) can harbor both, HSV-1 and HSV-2 [5–7]. Viral gene expression can be reactivated upon stress and a decline in the local immune responses, and progeny viruses are transported back to the periphery, where they cause lytic infections. Efficient targeting of alphaherpesviruses to the axons is essential for axonal spread and thus disease. Severe outcomes are potentially blinding *herpes keratitis* upon spread within the eyes or life-threatening *herpes encephalitis* upon spread within the brain [reviewed in 8,9].

The HSV-1 virion contains a large double-stranded DNA genome encased in a viral capsid, numerous tegument proteins and an envelope with many viral membrane proteins. Furthermore several host proteins and mRNAs have been detected in highly purified inocula [10–12]. HSV-1 assembly begins in the nucleus with genome packaging into capsids, which then traverse the nuclear envelope [reviewed in 13,14]. Cytosolic capsids associate with the inner tegument proteins pUL36 and pUL37 for their intracellular transport along microtubules to cytoplasmic membranes, where they meet other tegument and viral membrane proteins for secondary envelopment and virion formation [15–24]. In addition to pUL36 and pUL37, other structural proteins are required for efficient capsid envelopment. HSV-1 mutants lacking either pUL36 or pUL37, or the membrane proteins pUL20 or glycoprotein K (gK) are severely impaired in cytoplasmic envelopment, and accumulate cytosolic capsids instead [15,16,18,20,25–32]. HSV1-pUL20 and gK form functional complexes that connect with the capsids via pUL37 which in turn binds to pUL36 and the small capsid protein VP26 [33–36]. Another prominent tegument linker is VP16, with binding sites for pUL36, VP11/12, VP22 and gH [22,34,37–39]. VP22 in turn can bind ICP0, pUL16, gD, gE and gM [reviewed in 21,22]. The resulting vesicles are transported to the cell periphery and fuse with the plasma membrane to release infectious virions [reviewed in 13,14,22].

HSV-1 cytosolic capsids and complete virions within transport vesicles are targeted from the neuronal somata to axons to a varying extent; this has led to different hypotheses on the mode of neuronal alphaherpesvirus assembly [reviewed in 23,40,41]. According to the *married model*, capsid envelopment occurs exclusively in the soma, and only transport vesicles harboring complete virions enter axons. The *separate model* refers to different cargo structures being targeted independently of each other to the axons; namely capsids with associated tegument proteins as well as vesicles harboring viral envelope proteins and tegument proteins associated to their cytosolic tails.

Many structural proteins contribute to the neuronal spread of alphaherpesviruses, but the molecular determinants that are required for microtubule motor recruitment and for targeting from the soma to the axon gate have not been fully dissected. Purified HSV-1 capsids with inner tegument proteins can recruit the microtubule motors kinesin-1, kinesin-2, dynein and its cofactor dynactin to their surface, are translocated along microtubules *in vitro*, and can dock to nuclear pores [11,42–44]. pUL36 of HSV-1 and of the porcine pseudorabies virus (PRV) harbor potential binding motives for kinesin-1 light chains, and PRV-pUL36 has been shown to bind dynein and dynactin [45–47]. For these reasons and based on many additional functional studies, pUL36 and pUL37 are considered the most likely candidates for microtubule motor recruitment [reviewed in 23,45,46,48].

We therefore hypothesized that pUL36 and pUL37 might be sufficient for axonal capsid targeting and axonal capsid transport. As capsid envelopment may be a fast process, and since therefore the steady-state concentration of cytosolic capsids might be low, we used HSV-1 mutants to enrich for assembly intermediates. We infected neurons with HSV1-ΔUL20, HSV1-ΔUL37 or HSV1-ΔUL36 that accumulated cytosolic capsids decorated with pUL36 and pUL37, only with pUL36, or lacking both. Our previous data indicate that acquisition of both pUL36 and pUL37 is essential to enable capsids to enlist microtubule transport in epithelial cells [20]. However, while these inner tegument proteins enabled intracellular capsid motility of HSV1-ΔUL20 in epithelial cells and the somata of DRG neurons, they did not suffice to target cytosolic capsids to the axons. Our data indicate that HSV-1 particles have to acquire additional tegument and envelope proteins, and suggest that cytoplasmic envelopment needs to be completed prior to axonal transport which is in accordance with the *married model* for assembly of alphaherpesviruses in neurons.

## Results

### HSV-1 infection of mature neurons from dorsal root ganglia of adult mice

To investigate HSV-1 axonal targeting, we cultured primary neurons derived from dissociated DRG of adult mice until they had developed mature neurites. Within 3 to 5 days of culture *in vitro* (div), the neurons expressed the axonal microtubule-associated protein tau (not shown), phosphorylated neurofilament, un-phosphorylated neurofilament, and ankyrinG. In the somata, there were short β-III-tubulin microtubules and careful analysis often revealed a perinuclear microtubule-organizing center (S1Aii Fig, arrow), but individual microtubules could not be discerned in the neurites (S1A Fig). There were less phosphorylated neurofilament H and M in the somata than in the neurites (S1B Fig), while non-phosphorylated neurofilament H epitopes were distributed more evenly (S1C Fig), as reported for rat nervous tissue [49]. Likewise ankyrinG, another axonal marker was targeted to the neurites (S1D Fig). *In situ*, peripheral sensory axons also contain ankyrinG along the entire axons up to the dermal-epidermal junction [50].

To evaluate the microtubule polarity in these neurites, we transduced the neurons at 1 div with a lentiviral vector expressing end-binding protein 3 (EB3), which associates with dynamically growing microtubule plus-ends [51]. At 4 div, live-cell imaging as well as time-projections showed that all EB3-GFP comets moved away from the soma, suggesting that most, if not all, microtubule plus-ends faced towards the distal ends of the neurites (not shown). These results indicate that the DRG neurons had regrown neurites with axonal features and with a unipolar microtubule polarity. We have shown previously that such neurons are productively infected, and that they transmit HSV-1 to co-cultured epithelial cells upon infection with the HSV1(17^+^)Lox-Che (c.f. Table 1), which had been cloned into a bacterial artificial chromosome (BAC), and which expresses mCherry as a reporter [52,53].

**Table 1:** HSV1(17^+^)Lox strains used in this study.

HSV1(17^+^)Lox	Genotype	Reference
-CheVP26	codons for aa1-7 of VP26 replaced with mCherry	[20]
-ΔUL36	entire coding sequence of UL36 deleted, no pUL36 expression	[20,47]
-ΔUL37	codons for aa1-1121 of UL37 replaced by FRT site, no pUL37 expression	[20]
-ΔUL20	pUL20 start codon mutated and stop codons inserted, no pUL20 expression	this study
-CheVP26-ΔUL36	codons for aa1-7 of VP26 replaced with mCherry, entire coding sequence of UL36 deleted, no pUL36 expression	[20,47]
-CheVP26-ΔUL37	codons for aa1-7 of VP26 replaced with mCherry, codons for aa1-1121 of UL37 replaced by FRT site, no pUL37 expression	[20]
-CheVP26-ΔUL20	codons for aa1-7 of VP26 replaced with mCherry, pUL20 start codon mutated and stop codons inserted, no pUL20 expression	this study

### HSV-1 mutants lacking pUL36, pUL37, or pUL20 assemble different cytosolic capsids

To generate cytosolic capsids with different tegument protein composition, and thus to determine whether the requirements for intracellular capsid motility parallel those for axonal targeting, we constructed HSV1(17^+^)Lox-CheVP26-ΔUL36 [20], -CheVP26-ΔUL37 [20], and -CheVP26-ΔUL20 (this study) in the same genetic background (c.f. Table 1). We deleted the ATG start codon and a second ATG, and inserted three stop codons into the UL20 gene, since it includes the promotor of UL19 that codes for the major capsid protein VP5. Restriction analyses of pHSV1(17^+^)Lox-ΔUL20 and -CheVP26-ΔUL20 indicated the addition of mCherry to VP26 (*AscI* and *BamHI*, C in S2A Fig), and that a resistance gene inserted with the UL20 mutations had been removed (*XhoI*, asterisks in S2A Fig). Further *HindIII* (S2A Fig), *EcoRI*, or *EcoRV* digestions resulted in the expected fragment sizes (not shown). HSV1(17^+^)Lox-ΔUL20 and -CheVP26-ΔUL20 were recovered by transfecting the corresponding BACs into Flp-In-CV-1-cells that express pUL20 *in trans* [54]. Sequencing of the mutated region confirmed the introduction of the intended changes (not shown). The lack of the ATGs and the introduced stop codons prevented the expression of pUL20, whereas pUL37 expression was unchanged (S2B Fig). The intra- and extracellular titers of HSV1(17^+^)Lox-ΔUL20 and -CheVP26-ΔUL20 were about 1,000-fold lower than their parental strains in non-complementing Vero cells, but higher in a pUL20 complementing cell line (S2C and S2D Fig). Similar results have been reported for HSV1-ΔUL20 mutants in other genetic backgrounds [25,26,54–56]. There were little differences between the parental Lox and the -CheVP26 strains, indicating that tagging VP26 with mCherry (Che) did not impair HSV-1 replication, as reported before [24,57].

**Fig 1:**
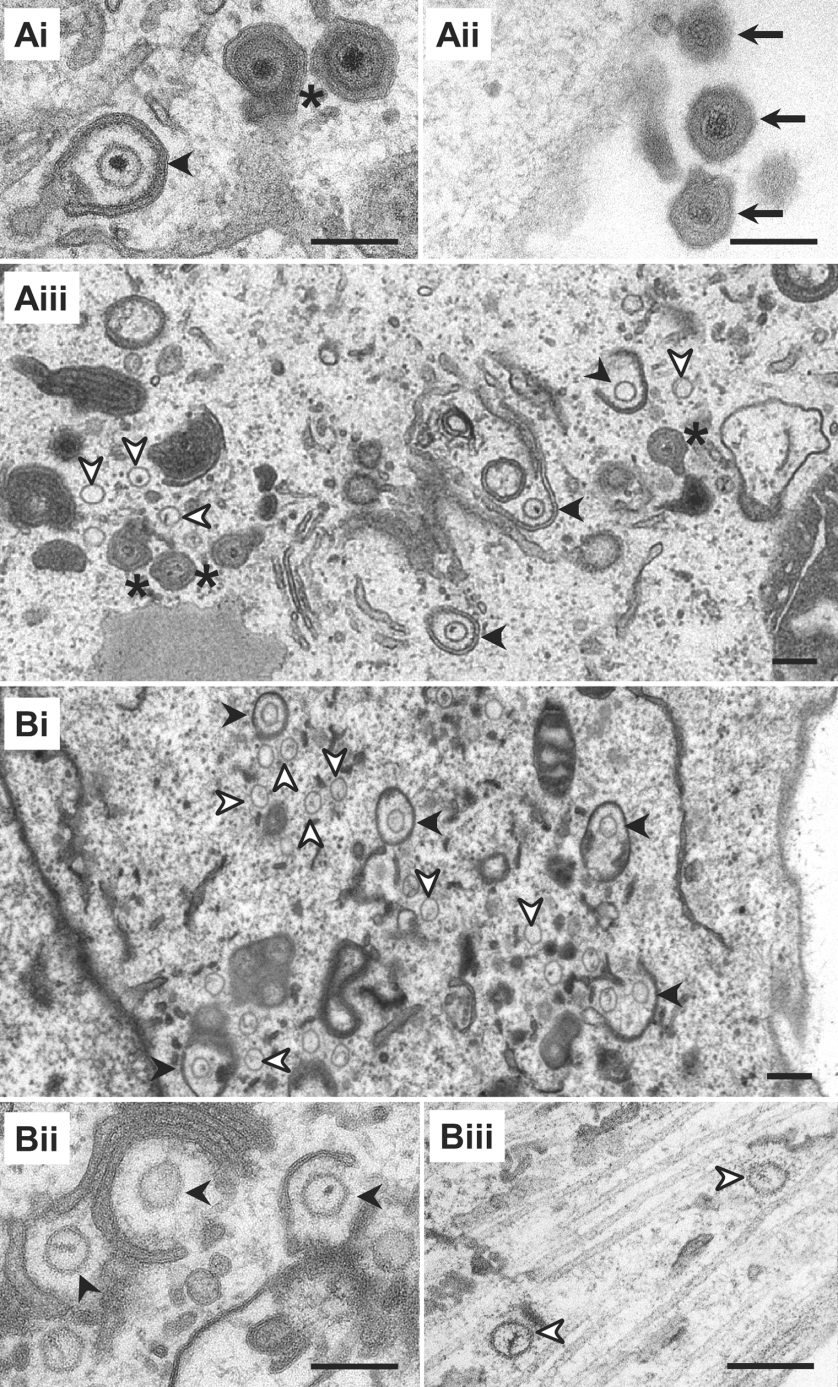
HSV1-pUL20 is required for secondary envelopment in epithelial cells. Vero cells infected with 1.9 x 10^6^ pfu/mL (10 pfu/cell) of HSV1(17^+^)Lox (A), -ΔUL20 (Bi and Bii), or -CheVP26-ΔUL20 (Biii), fixed at 14 hpi, and processed for conventional electron microscopy. Cytosolic capsids (white arrowheads), wrapping intermediates with capsids being closely associated with cytoplasmic membranes (black arrowheads), virions after complete secondary envelopment (asterisk), and extracellular virions (arrow). Scale bars are 200 nm.

**Fig 2:**
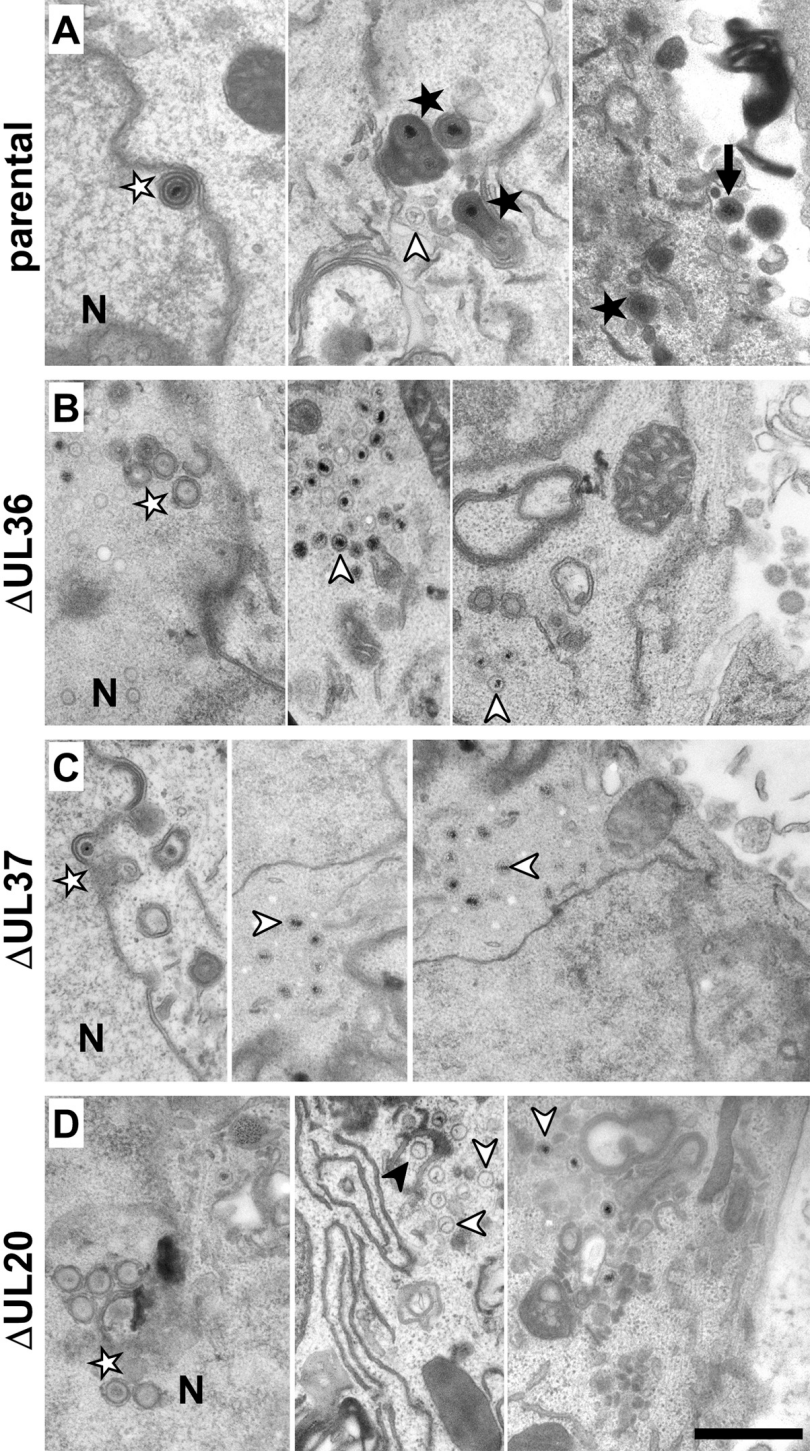
Cytosolic capsids accumulate in the absence of pUL36 or pUL37 and pUL20 is required for secondary envelopment in neurons. DRG neurons infected with 1 x 10^7^ pfu/mL of HSV1(17^+^)Lox-CheVP26 (A), -ΔUL36 (B), -ΔUL37 (C), -ΔUL20 (D), fixed at 24 or 30 hpi and processed for conventional electron microscopy. Nucleus (N), primary enveloped virions (white stars), cytosolic capsids (white arrowheads), wrapping intermediates with capsids being closely associated with cytoplasmic membranes (black arrowheads), virions after complete secondary envelopment (black star), and extracellular virions (arrow). Scale bar is 500 nm.

Using conventional electron microscopy, we next analyzed virus morphogenesis. Upon infection of Vero cells with HSV1(17^+^)Lox (Fig 1A) or -CheVP26 (not shown), viral particle maturation proceeded as expected with the formation of nuclear capsids, the appearance of cytosolic capsids, partially and completely enveloped cytoplasmic capsids, and extracellular virions associated with the plasma membrane. With secondary envelopment, capsids acquired an electron dense tegument layer, and the electron dense genomes were well preserved (Fig 1Ai and 1Aiii; asterisk). Therefore the morphology of capsids and tegument of virions inside authentic transport vesicles was more similar to that of extracellular virions (Fig 1Aii; black arrow) than to that of cytosolic capsids (Fig 2Aiii; white arrowhead) even if they were partially enveloped (Fig 1Ai and 1Aiii, black arrowhead). Nuclear capsid egress of the mutants HSV1(17^+^)Lox-ΔUL20 (Fig 1Bi and 1Bii) and -CheVP26-ΔUL20 (Fig 1Biii) was unaffected, resulting in many cytosolic capsids, and several of them were closely associated with cytoplasmic membranes (Fig 1Bi and 1Bii; black arrowhead). We detected neither capsids with the characteristic morphology of completed secondary envelopment nor virions bound to the plasma membrane in the absence of pUL20. Furthermore, in contrast to infection with HSV1(17^+^)Lox-ΔUL36 or -ΔUL37 [20], we did not detect any cytoplasmic or extracellular L-particles, which are viral envelopes with an electron dense tegument but lacking capsids [58–60].

Next, we analyzed DRG cultures infected with HSV1(17^+^)Lox-CheVP26, -CheVP26-ΔUL36, -CheVP26-ΔUL37, -CheVP26-ΔUL20 (Fig 2) or their respective untagged strains (not shown) by electron microscopy. Neurons identified by the characteristic morphology of their nuclei and infected with the parental HSV-1 strains contained nuclear capsids, primary envelopment intermediates (Fig 2A, white star), cytosolic capsids (Fig 2A, white arrowhead), and enveloped virions (Fig 2A, black star). Neurons infected with HSV1(17^+^)Lox-CheVP26-ΔUL36 (Fig 2B), -CheVP26-ΔUL37 (Fig 2C), or -CheVP26-ΔUL20 (Fig 2D) contained non-enveloped cytosolic capsids (Fig 2, white arrowhead), but did not reveal any extracellular virions bound to their plasma membranes. HSV1(17^+^)Lox-CheVP26-ΔUL20 infected neurons contained in addition wrapping intermediates (Fig 2D, black arrowhead). A quantification of the number of cytoplasmic HSV-1 assembly intermediates revealed that cells infected with HSV1(17^+^)Lox-ΔUL20 or -CheVP26-ΔUL20 had a similar ratio of cytosolic capsids to membrane-associated capsids as upon infection with the respective parental strains HSV1(17^+^)Lox or -CheVP26 (Table 2).

**Table 2:** HSV1(17^+^)Lox-ΔUL20 is impaired in secondary envelopment.

Cell Type	HSV1(17^+^) type	No. of cells	Area [μm^2^]	No. of cytoplasmic capsids	No. of capsids/area [1/1000 μm^2^]	% of cytoplasmic capsids		
						Cytosolic	Wrapping intermediates	Enveloped virions
Vero cells	Lox	39	3958	269	68	48	39	13
	Lox-CheVP26	39	4294	308	72	48	41	11
	Lox-ΔUL20	38	4158	369	89	53	47	0
	Lox-CheVP26-ΔUL20	35	4318	817	189	54	46	0
neurons	Lox	28	324	48	148	27	35	37
	Lox-CheVP26	31	347	92	265	46	44	10
	Lox-ΔUL20	18	208	108	519	52	48	0
	Lox-CheVP26-ΔUL20	19	220	58	263	47	53	0

Vero cells or DRG neurons infected with HSV1(17^+^)Lox, Lox-mCheVP26, Lox-ΔUL20, or Lox-CheVP26-ΔUL20 were analyzed by electron microscopy, and the number of cytosolic capsids, wrapping intermediates and enveloped virions were determined.

**Fig 3:**
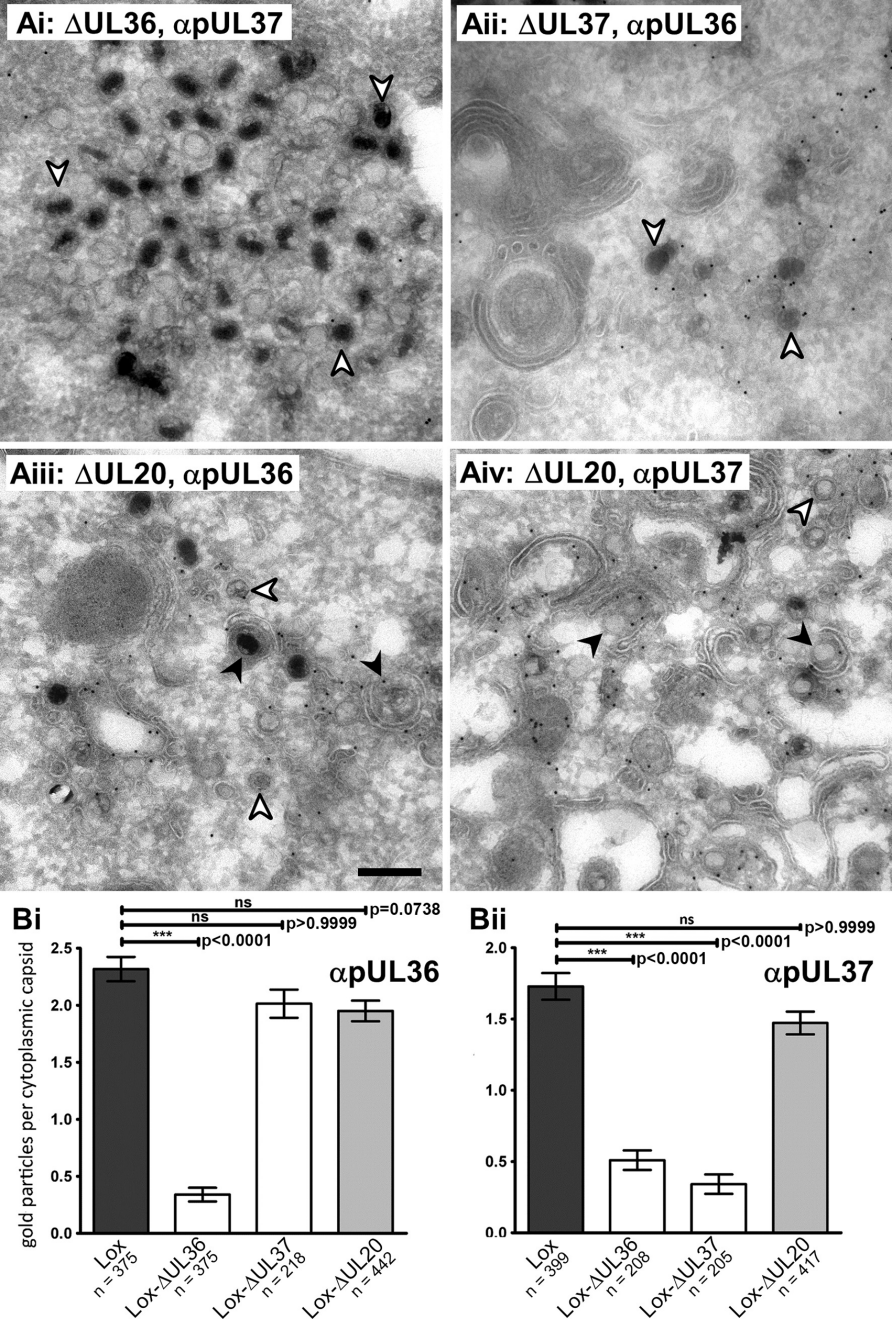
In the absence of pUL20, HSV1 capsids associate with pUL36 and pUL37. (A) Vero cells were infected with 1.1 x 10^7^ pfu/mL (10 pfu/cell) of HSV1(17^+^)Lox-ΔUL36 (i), -ΔUL37 (ii) or -ΔUL20 (iii and iv). At 16 hpi, the cells were fixed and processed for immunoelectron microscopy with antibodies against pUL36 (ii and iii) or pUL37 (i and iv). (B) Immunogold labeling of cytoplasmic capsids was quantified after labeling with anti-pUL36 (Bi) or anti-pUL37 (Bii). The mean values and standard errors of the mean (SEM) were calculated. P-values were calculated with the Kruskal-Wallis test and Dunn’s multiple comparison test.

To characterize the surface protein composition of the cytosolic capsids, Vero cells were infected with HSV1(17^+^)Lox or -ΔUL20 for 16 h, fixed, and ultrathin cryosections were prepared for quantitative immunoelectron microscopy. We have reported similar experiments for -ΔUL36 and -ΔUL37 before [20], and show their results here again for a direct comparison (Fig 3Ai and 3Aii). Cytosolic capsids of both, Lox [20] and -ΔUL20 were labeled with anti-pUL36 (Fig 3Aiii) and anti-pUL37 (Fig 3Aiv). There were on average 2.2 gold particles per capsid for HSV1(17^+^)Lox, 2 for -ΔUL37 and -ΔUL20, but only 0.5 for -ΔUL36 (Fig 3Bi) using anti-pUL36 antibodies. Upon labeling with anti-pUL37, there were 1.7 or 1.5 gold particles per capsid for HSV1(17^+^)Lox or -ΔUL20, but only 0.5 for -ΔUL36 and -ΔUL37 (Fig 3Bii). These results indicate that cytoplasmic capsids of Lox-ΔUL20 had recruited pUL36 and pUL37 to a similar extent as those of the parental HSV1(17^+^)Lox, while capsids of -ΔUL37 lacked pUL37 but still recruited pUL36, and capsids of -ΔUL36 lacked both, pUL36 and pUL37. Thus, the HSV1(17^+^)Lox-ΔUL36, -ΔUL37, and -ΔUL20 as well as HSV1(17^+^)Lox-CheVP26-ΔUL36, -ΔUL37, and -ΔUL20 mutants generated cytosolic capsids exposing different proteins on their surface, namely no inner tegument, only pUL36, or both pUL36 and pUL37.

### HSV-1-pUL20 is dispensable for capsid motility in epithelial cells and the somata of neurons

**Fig 4:**
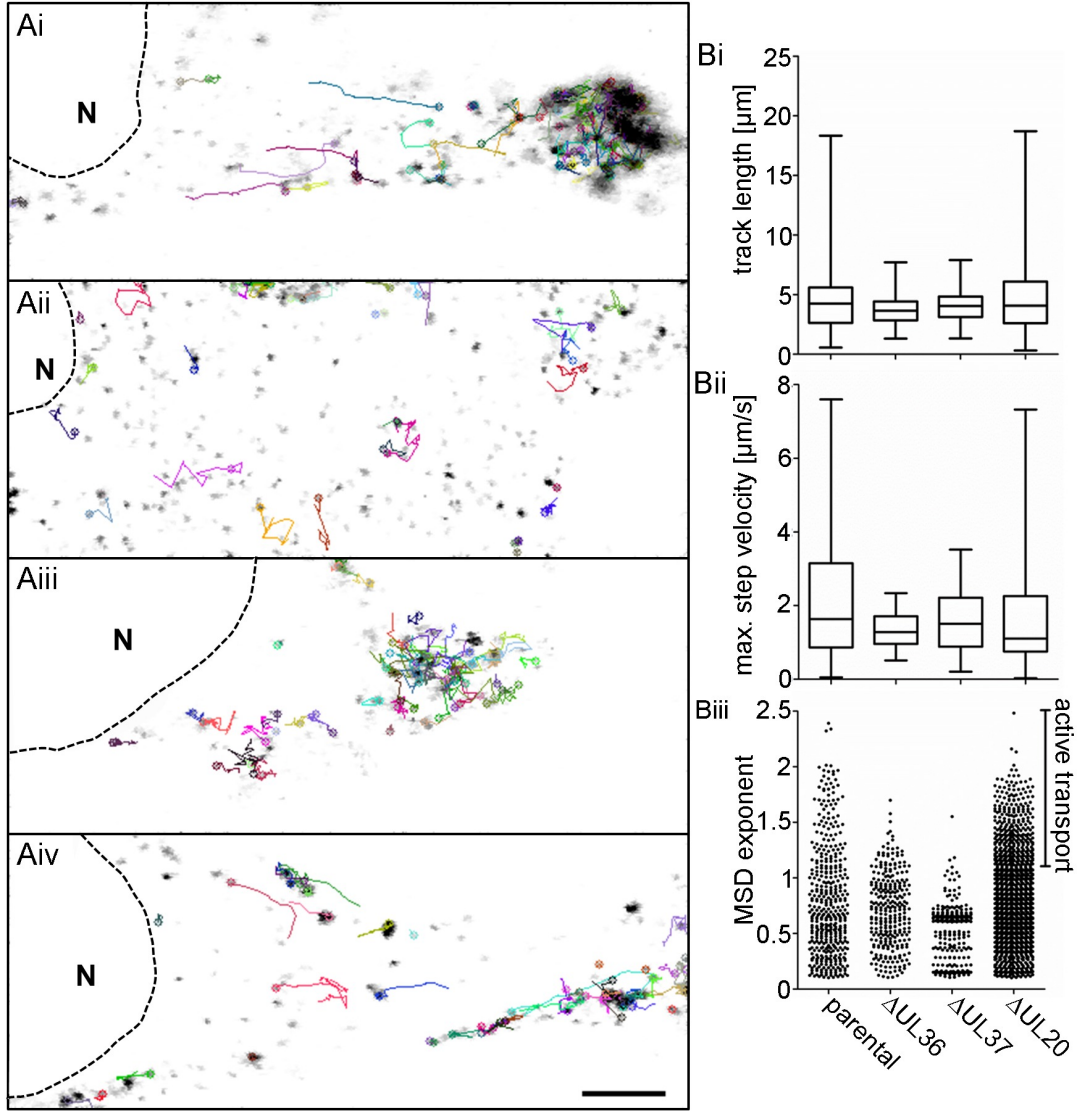
HSV1-pUL20 is not required for directed intracellular transport in epithelial cells. Vero cells were infected with 6.4 x 10^6^ pfu/ml (10 pfu/cell) of HSV1(17^+^)Lox-mCheVP26 (Ai), -ΔUL36 (Aii), -ΔUL37 (Aiii), or -ΔUL20 (Aiv) and movies were acquired at 8–10 hpi. (A) Stills of movies in Vero cells (S1–S4 Movies) and with representative track profiles (indicated by a colored line and the front of each track marked by an open circle). Scale bar, 5 μm. (B) Analysis of track profiles of HSV1(17^+^)Lox-mCheVP26 (parental), -ΔUL36, -ΔUL37, or –ΔUL20. (Bi) Track length and (Bii) maximum step velocity of tracks with a MSD_ex_ ≥ 1.2, (Biii) mean square displacement exponent (MSD_ex_), with each dot representing one track. Box and whiskers with min and max.

To characterize the intracellular capsid motility in the presence of different inner tegument proteins, we infected Vero cells with HSV1(17^+^)Lox-CheVP26, -CheVP26-ΔUL36, -CheVP26-ΔUL37, or -CheVP26-ΔUL20 and acquired confocal fluorescence time-lapse movies with a temporal resolution of 5 images per second. For a direct comparison, we analyzed in parallel movies with -CheVP26 and -CheVP26-ΔUL20 recorded in this study as well as movies that we had recorded with -CheVP26-ΔUL36 and -CheVP26-ΔUL37 previously and published in Sandbaumhüter et al. [20]. The time-projections of the tracks show that capsids of Lox-CheVP26 (Fig 4Ai) and -CheVP26-ΔUL20 (Fig 4Aiv) exerted short and long range transport towards the nucleus and towards the cell periphery (S1 and S4 Movies). In contrast, the tracks of -CheVP26-ΔUL36 (Fig 4Aii) and -CheVP26-ΔUL37 (Fig 4Aiii) revealed only random, undirected motility (S2 and S3 Movies).

While the average track length (Fig 4Bi) and the maximum step velocity (Fig 4Bii) were similar for the capsids of -CheVP26, -CheVP26-ΔUL36, -CheVP26-ΔUL37, and -CheVP26-ΔUL20, there were more tracks with a length of more than 5 μm and with a velocity exceeding 1 μm/s for the capsids of the parental Lox-CheVP26 and the -CheVP26-ΔUL20 than for the capsids of -CheVP26-ΔUL36 and -CheVP26-ΔUL37. We furthermore determined the mean square displacement exponent (MSD_ex_) for each track (Fig 4Biii). The MSD_ex_ is a characteristic feature for the displacement mode of a single motile particle from its starting position over time. The MSD is defined as the square of the travelled distance from the starting point of the track, which is calculated for each time point, and plotted against the time. The slope of such a curve in a log-log plot defines the MSD_ex_ and indicates whether a particle has undergone active transport (MSD_ex_ > 1), free diffusion (MSD_ex_ = 1), or confined diffusion [MSD_ex_<1; 61,62,63]. While the MSD_ex_ of the majority of the tracks denoted free or confined diffusion as expected for any intracellular cargo, the MSD_ex_ was greater than 1 for more tracks of Lox-CheVP26 and -CheVP26-ΔUL20 than for -CheVP26-ΔUL36 or -CheVP26-ΔUL37.

**Fig 5:**
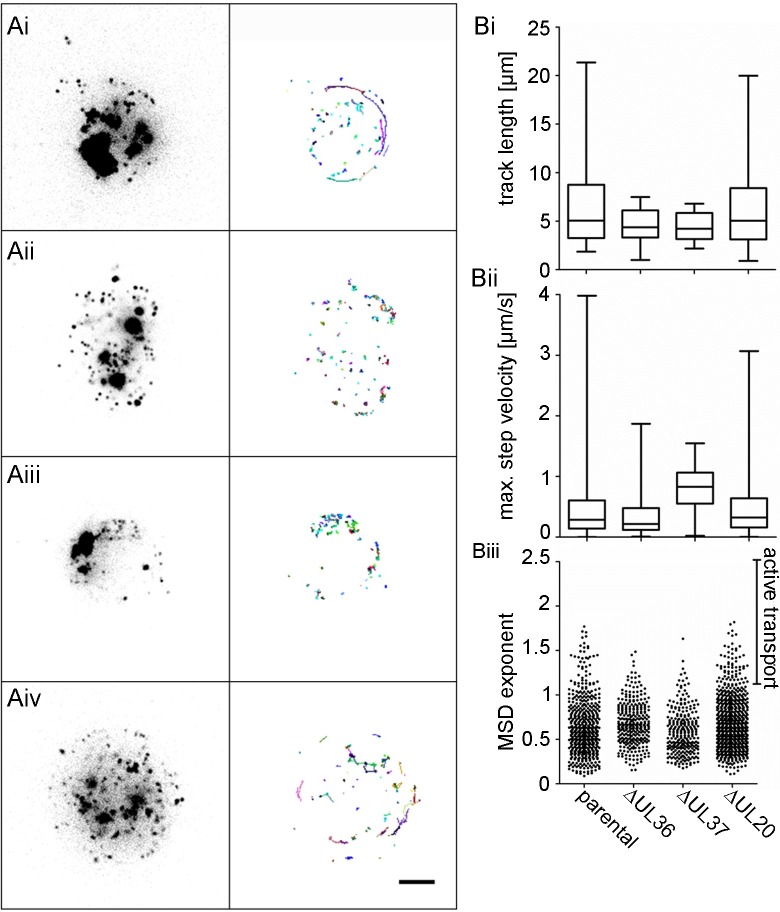
HSV1-pUL20 is not required for directed intracellular transport in neurons. DRG neurons infected at 5 div with 1 x 10^7^ pfu/mL of HSV1(17^+^)Lox-CheVP26 (Ai), -ΔUL36 (Aii), -ΔUL37 (Aiii), or -ΔUL20 (Aiv) and movies were acquired between 22 and 31 hpi. (A) Stills of movies in DRG neurons (S5–S8 Movies) with representative track profiles (indicated by a colored line and the front of each track marked by an open circle) of HSV1(17^+^)Lox-mCheVP26 (i, parental), -ΔUL36 (ii), -ΔUL37 (iii), or -ΔUL20 (iv). Scale bar, 5 μm. (B) DRG neurons: Analysis of track profiles of HSV1(17^+^)Lox-mCheVP26 (parental), -ΔUL36, -ΔUL37, or -UL20. (Bi) Track length and (Bii) maximum step velocity of tracks with a MSD_ex_ ≥ 1.2, (Biii) mean square displacement exponent (MSD_ex_) with each dot representing one track. Box and whiskers with min and max.

We furthermore infected DRG neurons with HSV1(17^+^)Lox-CheVP26, -CheVP26-ΔUL36, -CheVP26-ΔUL37, or -CheVP26-ΔUL20 and acquired spinning disk microscopy time lapse movies of the cell bodies with a temporal resolution of 20 images per second. Similar as in the epithelial cells, capsids of the parental (Fig 5Ai; S5 Movie) and -CheVP26-ΔUL20 (Fig 5Aiv; S8 Movie) were transported over short and longer distances as indicated by the time projection lines. In contrast, the capsids of -CheVP26-ΔUL36 (Fig 5Aii; S6 Movie) and -CheVP26-ΔUL37 (Fig 5Aiii, S7 Movie) only moved in an undirected fashion. Quantification revealed that the transport characteristics in DRG neuronal cell bodies were the same as in epithelial cells (Fig 5Bi to 5iii). Thus, the motility features of the cytosolic capsids of -CheVP26-ΔUL36 or -CheVP26-ΔUL37 in epithelial cells and sensory neurons were indicative for intracellular diffusion which is also the mode of motility in the absence of microtubules after nocodazole treatment [62]. In contrast, the capsids of -CheVP26 and -CheVP26-ΔUL20 had transport characteristics typical for directed, active microtubule-dependent transport.

### HSV-1 axonal targeting in the absence of pUL36, pUL37, or pUL20

**Fig 6:**
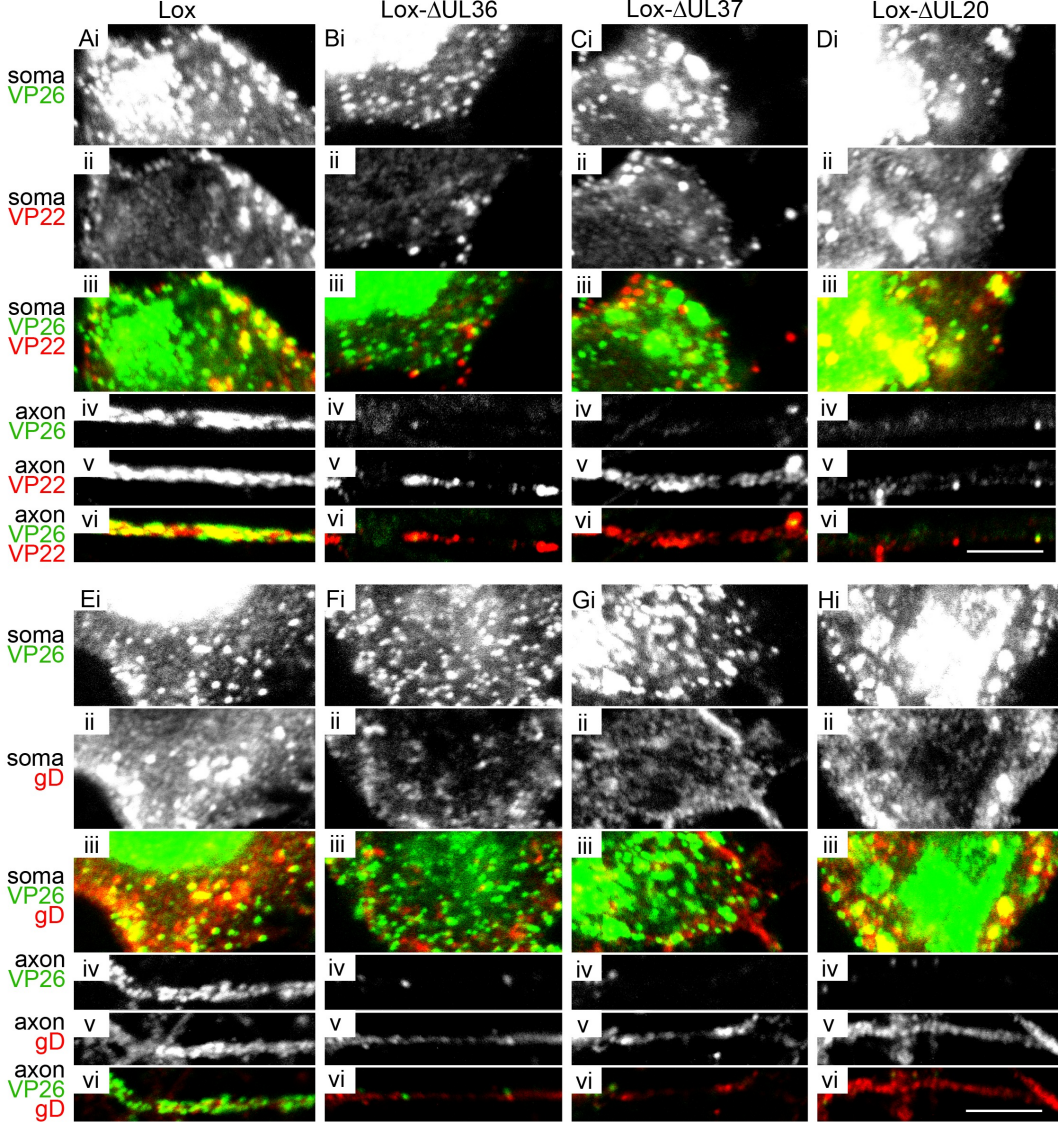
HSV1-pUL36, pUL37 and pUL20 are required for efficient targeting of capsids to axons. DRG neurons were infected after 3 div with 1 x 10^7^ pfu/mL of HSV1(17^+^)Lox (A and E), -ΔUL36 (B and F), -ΔUL37 (C and G), or -ΔUL20 (D and H), fixed and permeabilized using the PHEMO protocol at 26 hpi, labeled with antibodies directed against VP26 (pAb VP26_aa95-112_) and VP22 (mAb 22–3, A-D) or gD (mAb DL6, E-H), and analyzed by confocal fluorescence microscopy. (i and iv) anti-VP26, (ii and v) anti-VP22 in the upper panel and anti-gD in the lower panel, (iii and vi) anti-VP26 in green and anti-VP22 in the upper panel and anti-gD in the lower panel in red. (i-iii) Cell bodies with nucleus and cytoplasm; (iv-vi) proximal axons. Scale bar, 5 μm.

**Fig 7:**
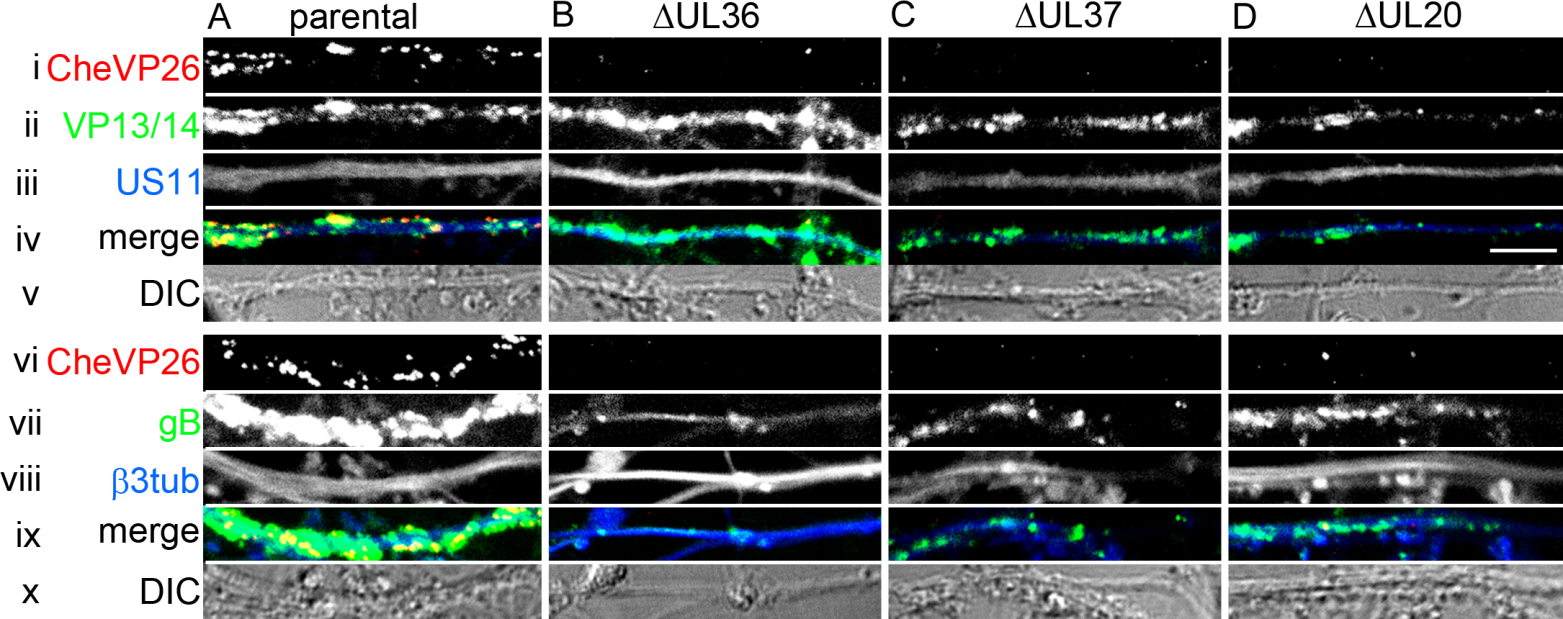
HSV1 outer tegument and envelope proteins are targeted to the axon independent of capsids. DRG neurons were infected after 3 div with 1 x 10^7^ pfu/mL of HSV1(17^+^)Lox-CheVP26 (A), -ΔUL36 (B), -ΔUL37 (C), or -ΔUL20 (D), fixed and permeabilized using the PHEMO protocol at 24 hpi, labeled with antibodies directed against VP13/14 (R220, ii), gB (R69, vi) or β-III-tubulin (mAb 5564, vii) and the axons were analyzed by confocal microscopy. CheVP26 (i and v), merge (iii and viii), differential interference contrast image (DIC, iv and ix). Scale bar is 5 μm.

After we had characterized the intracellular motility of capsids in the presence of pUL36 and pUL37 (-ΔUL20), of only pUL36 (-ΔUL37), or lacking both (-ΔUL36), we asked which capsid types could be targeted to the axonal outlet. We infected primary DRG neurons with HSV1(17^+^)Lox, -ΔUL36, -ΔUL37, or ΔUL20 (Fig 6) or the respective HSV1(17^+^)Lox-CheVP26 strains (S3 Fig, Fig 7). At 24 to 26 hpi, the cells were fixed, permeabilized and labeled with antibodies directed against the capsid protein VP26 (Fig 6i and 6iv panels, green in 6iii), and the tegument proteins VP22 (Fig 6A–6D, ii and v panels, red in iii and vi), the tegument protein VP13/14 (Fig 7, ii panels, green in iii), the envelope protein gD (Fig 6E–6H, ii and iv panels, red iii and vi), the envelope protein gB (Fig 7, vi panels, green in viii), or the neuron specific beta-3-tubulin (Fig 7vii). Representative images from at least 3 independent experiments for the different strains are shown in Figs 6, S3 and 7.

After infection with HSV1(17^+^)Lox (Fig 6A and 6E) or HSV1(17^+^)Lox-CheVP26 (Figs S3A and 7A), the nucleoplasm (Figs 6Ai, 6Ei and S3Ai), the cytoplasm (Figs 6Ai, 6Ei and S3Ai) and the axons (Fig 6Aiv, 6Eiv and 7A) were filled with capsids; in the cytoplasm, the capsids were often clustered. Infection with -ΔUL36 (Figs 6B, 6F, S3B and 7B) resulted in a dispersed and more random distribution of capsids in the cytoplasm of the somata (S3B Fig), with fewer capsids being targeted to the axons (Figs 6Biv, 6Fi, 6Fv and 7B). After infection with -ΔUL37 (Figs 6C, 6G, S3C and 7C), capsids clustered within the cytoplasm and the cell morphology was often altered; again very few capsids had been targeted to the axons (Figs 6Civ, 6Giv, 7Ci and 7Cv). The stronger cytopathic effects as indicated by membrane blebbing upon infection upon infection with the HSV1-ΔUL37 strains (S3C Fig) might be due to the lack of pUL37 targeting RIG-I and blocking RNA-induced activation [64], but we did not investigate this further.

Unexpectedly, there were also only very few axonal capsids upon infection with -ΔUL20 (Figs 6Hiv and 7D). The capsid distribution of -ΔUL20 in the somata was similar to that of the parental HSV1(17^+^)Lox, but with reduced accumulation at the plasma membrane (Fig 6Div, S3Di). After infection with -ΔUL36, -ΔUL37, or -ΔUL20, the outer tegument proteins VP22 (Fig 6Bv, 6Cv and 6Dv) and VP13/14 (Fig 7Bii, 7Cii and 7Dii) as well as gD (Fig 6Fv, 6Gv and 6Hv) and the envelope proteins gB (Fig 7Bvi, 7Cvi and 7Dvi) had still been targeted to the axons as for the parental strain (Figs 6A, 6E and 7A), indicating that axonal transport *per se* had not been impaired. Nevertheless, the deletion mutants -ΔUL36, -ΔUL37, and -ΔUL20 were incapable of efficient axonal capsid transport.

**Fig 8:**
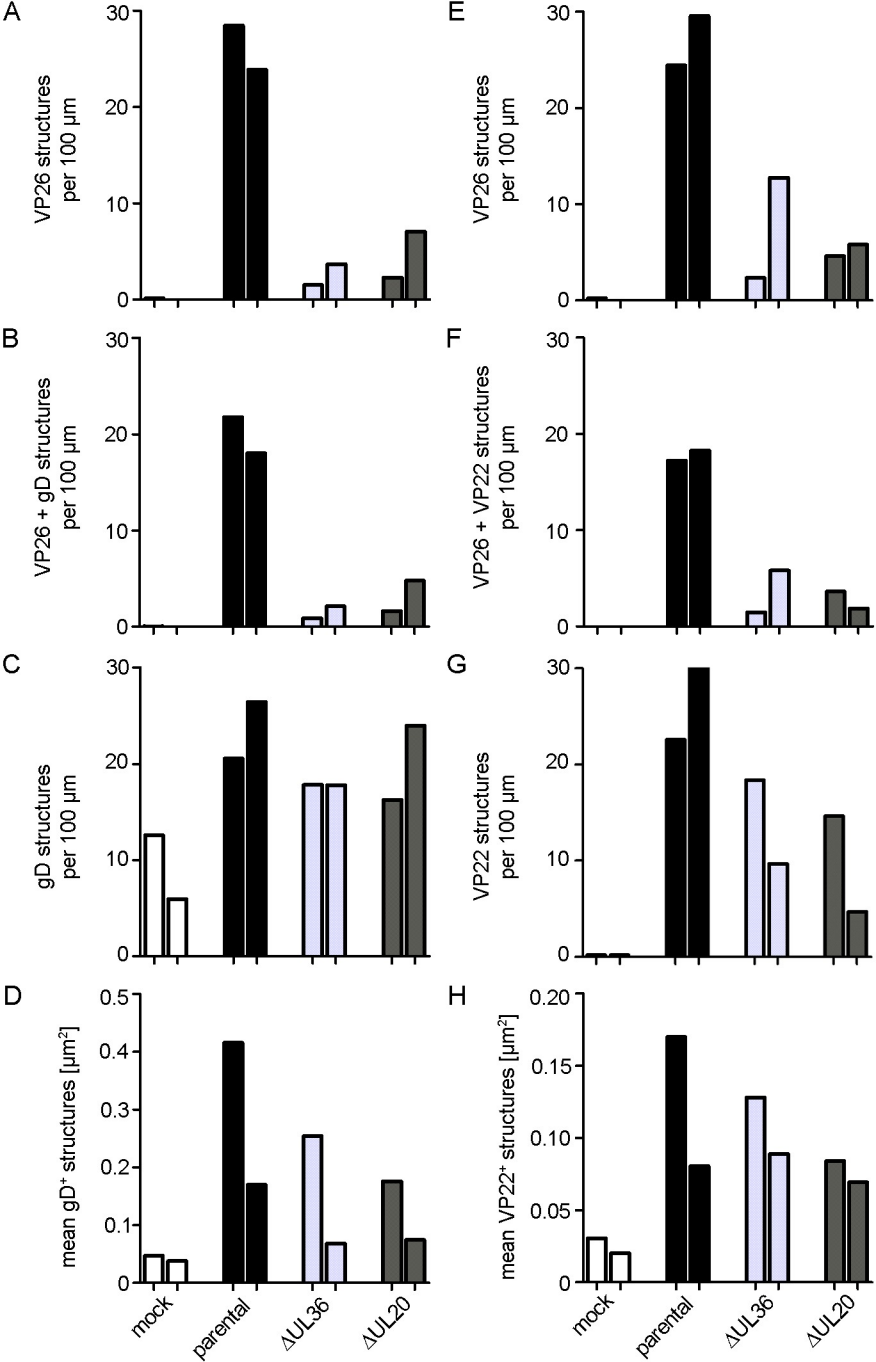
HSV1-pUL36, pUL37 and pUL20 are required for efficient targeting of capsids to axons. DRG neurons were infected after 3 div with 1 x 10^7^ pfu/mL of HSV1(17^+^)Lox, Lox-ΔUL36, Lox-ΔUL37, or Lox-ΔUL20 (or mock treated, fixed and permeabilized using the PHEMO protocol at 24 to26 hpi, labeled with antibodies directed against VP26 (pAb VP26_aa95-112_) and gD (mAb DL6, A-D) or VP22 (mAb 22–3, E-H), and analyzed by confocal fluorescence microscopy. The images were quantified with a semi-automated algorithm. Each bar represents the mean from one experiment, normalized to the parental values. (A-D) Labeling with anti-VP26 and anti-gD: (A) Total number of VP26 positive structures. (B) VP26 and gD double positive structures. (C) total gD positive structures (D) Size of gD-positive structures. (E-H) Labeling with anti-VP26 and anti-VP22: (E) Total VP26 positive structures. (F) Total number of structures labeled for VP26 and VP22. (G) Total number of structures labeled for gD. (D) Area of structures labelled for gD. The number of capsids, gD or VP22 structures, image numbers and total axon length are shown in S1 Table.

For quantitation, we scaled up two experiments to image random axonal regions and determined the number of different viral structures per axon length using a novel automated image analysis algorithm (S4 Fig). We determined the imaged axon length, the total number of capsids as detected by anti-VP26, and the fraction of these capsids colocalizing with gD or VP22 (c.f. S1 Table). After infection with the parental strain HSV1(17^+^)Lox, we counted 20 to 30 capsids per 100 μm axon length (Fig 8A; total VP26, black bars) of which 75% colocalized with gD (Fig 8B; VP26 + gD). Furthermore, there was a similar number of membrane structures containing gD and colocalizing with VP26 or not (Fig 8C, gD); the size of these structures varied considerably (Fig 8D). After infection with Lox-ΔUL36 (Fig 8; light grey columns), or -ΔUL20 (Fig 8; dark grey columns), there were only few capsids targeted to the axons (Fig 8A), which again mostly co-localized with gD (Fig 8B). However, the number of gD-containing membrane structures was only moderately reduced for the HSV-1 mutants suggesting that axonal transport *per se* had not been inhibited (Fig 8C). In uninfected neurons, there was also some anti-gD background signal (Fig 8C, mock), but these cross-reacting structures were much smaller (Fig 8D). We also quantified the number of capsids detected by anti-VP26 (Fig 8E), and the fraction of these capsids colocalizing with the outer tegument protein VP22 (Fig 8F) of a parallel set of samples. After infection with the parental strain HSV1(17^+^)Lox, we counted again 20 to 30 capsids per 100 μm axon length (Fig 8E; total VP26, black bars), of which 60% to 70% colocalized with VP22 (Fig 8F; VP26 + VP22). Furthermore, there was a similar number of structures containing VP22 colocalizing with VP26 or not (Fig 8G; VP22) which also varied considerably in size (Fig 8H). After infection with Lox-ΔUL36, or -ΔUL20, again fewer capsids had been targeted to the axons (Fig 8E), of which almost 80% co-localized with VP22 (Fig 8F). The number of VP22 containing structures was moderately reduced for the HSV-1 mutants supporting the notion that axonal transport *per se* had not been inhibited (Fig 8G). In uninfected neurons, there was almost no anti-VP22 background signal (Fig 8G, mock), and again these cross-reacting structures were much smaller (Fig 8H).

These experiments show that the inner tegument proteins pUL36 and pUL37 on the capsids of -ΔUL20 were not sufficient for axonal targeting. Instead, association with membranes and outer tegument proteins, and thus possibly completion of secondary envelopment seem to be a prerequisite for efficient capsid targeting into the axons.

## Discussion

Animal models as well as cultured neurons have contributed tremendously to elucidating the mechanisms of viral neuroinvasion, spread within the nervous system, and peripheral recurrent diseases [1,2,23]. Various aspects of human diseases are mimicked in murine HSV-1 infection models [65]. For example, upon infection of the cornea, HSV-1 reaches the trigeminal ganglia and causes *herpetic keratitis*; infection of the flank skin establishes latency in the DRG, and results in zosteriform skin lesions upon reactivation [52,66–70]. To further characterize alphaherpesvirus axonal targeting, we investigated here HSV infection of primary neurons derived from the DRGs of adult mice, and developed novel image processing algorithms to determine the amount of different axonal assembly intermediates in an unbiased fashion. We infected such neurons with HSV-1 mutants that assemble cytosolic capsids lacking pUL36 and pUL37, capsids associated only with pUL36, or capsids that recruited both, pUL36 and pUL37 to determine whether these inner tegument proteins implicated in microtubule-mediated transport were sufficient for intracellular capsid motility in epithelial cells and the somata of neurons as well as for capsid targeting to axons. Our data indicate that in addition to acquiring pUL36 and pUL37, capsids needed to complete secondary envelopment to be efficiently targeted to axons, to spread from neurons to neighboring cells, and thus to cause recurrent diseases.

### HSV infection of primary neurons derived from adult mice

Embryonic and neonatal neurons dissociated from trigeminal ganglia, DRG, or superior cervical ganglia of chicken, mouse, rat, or human have been used to study axonal trafficking and egress of HSV particles [71–78]. However, primary cultures from embryonic tissue do not present optimal models for age-related changes in differentiation, physiology, or late-onset disease [79–81]. We and others [82] therefore used neurons from adult mice to complement results obtained by infecting adult animals with HSV-1 [74,83] and to obtain functional insights into the process of axonal targeting of HSV-1 viral structures. The neurons from the DRG of adult mice formed neurites with axonal features which contained ankyrinG and microtubules of uniform polarity with the plus-ends pointing towards the axon endings. Furthermore, we have shown previously that HSV1(17^+^)Lox strains that have been cloned into a bacterial artificial chromosome and therefore lack the Ori_L_ [84,85] infect such neurons and spread the infection to neighboring epithelial cells [52,53]. Hence murine adult DRG neurons provide a versatile system to study productive HSV-1 infection and transport in axons with unipolar microtubules *in vivo* and *in vitro*.

### Dissection of HSV-1 axonal structures

While there is a growing consensus that the swine alphaherpesvirus PRV relies on the *married model* for axonal transport in all neuron types, the picture is less clear for HSV-1. According to the *married model*, capsids are enveloped exclusively in the somata, and thus it would be sufficient to expose a host or a viral postal code on the cytosolic membrane surface to target vesicles harboring fully assembled virions to axons [reviewed in 23,40,41]. But for HSV-1 two types of axonal cargo, free cytosolic capsids as well as capsids surrounded by an envelope and a transport vesicle membrane, have been detected to a varying degree [71,73,76,78,86–88].

Complementary methods have been used to image viral assembly intermediates. Antibodies have less access to capsids associated with membranes than to cytosolic capsids [89,90]. While fluorescent protein tags circumvent this challenge, they may shift the relative abundance of different assembly intermediates [57,72,73,84,91–94]. Furthermore, the resolution limit of confocal microscopy is larger than a capsid diameter of 125 nm leading to an overestimation of capsid colocalization with membrane markers. On the other hand, conventional electron micrographs provide sufficient contrast to distinguish hexagonal capsids from membrane vesicles or fully enveloped capsids within transport vesicles but only upon sufficient heavy metal deposition and in very thin sections of 50 nm, which results in an underestimation of membrane-associated capsids [47,71,87]. In embryonic rat hippocampal neurons, many cytosolic HSV-1 capsids are indeed not associated with cytoplasmic membranes as shown by 3-dimensional cryoelectron tomography [88]. The latter is in fact the best imaging method for this question, but limited to specialized centers and not amenable to medium-throughput [95]. For these reasons, we developed novel image quantitation algorithms and novel mutants to characterize HSV-1 axonal targeting, and used both, fluorescent tagging and antibodies to detect the capsid protein VP26, in combination with antibodies against the tegument proteins VP22 and VP13/14, and the envelope proteins gD and gB.

### The inner tegument proteins pUL36 and pUL37 are not sufficient for axonal targeting

The *separate model* of alphaherpesvirus egress postulates that different subassemblies, namely cytosolic capsids as well as membrane vesicles conveying envelope proteins, are targeted to axons to be assembled in the axons or just prior to trans-synaptic spread or even during budding at the axonal plasma membrane [reviewed in 23,40,41]. Neuronal cargoes enlist the so-called *smart* motors that utilize stabilized microtubules which lead to the tiny outlets to dendrites or to axons [96,97]. One such *smart* motor is kinesin-1 that preferentially binds to de-tyrosinated and acetylated microtubules, modifications that are typical for axonal microtubules while others, such as kinesin-5 or kinesin-13 prefer tyrosinated microtubules [97,98]. Based on extensive genetic, biochemical and cell biology experiments, pUL36 and pUL37 are considered the most likely candidates for motor recruitment to cytosolic capsids [reviewed in 45,48]. Alphaherpesviruses lacking pUL36 or pUL37 are impaired in microtubule-mediated intracellular transport [15,16,18–20,30–32,99–101]. HSV-1 capsids exposing the inner tegument proteins pUL36 and pUL37 but not naked capsids translocate along microtubules *in vitro* and recruit kinesin-1 and kinesin-2 from a brain cytosolic extract [11,43]. Finally, pUL36 of HSV-1 and PRV contains binding sites for dynein and dynactin, and possibly the light chains of kinesin-1 [46,47].

We therefore generated HSV-1 mutants that assembled un-enveloped cytosolic capsids with different tegument proteins. Consistent with their proposed function to recruit microtubule motors, the capsids of HSV1-ΔUL20 harboring pUL36 and pUL37 but not the ones of HSV1-ΔUL36 or HSV1-ΔUL37 were capable of active transport in epithelial cells or the somata of DRG neurons. The capsids of HSV1-ΔUL20 therefore resemble capsids which recruit the *smart* motor kinesin-1 from a cytosolic brain extract *in vitro* [11]. Our data indicate that capsid-associated pUL36 and pUL37 were sufficient for microtubule transport, but not for efficient axonal targeting.

It is possible that the capsids of HSV1-ΔUL20 had been associated with a larger amount of outer tegument proteins covering potential motor-binding sites on the inner tegument. Capsids with lower amounts of outer tegument recruit more kinesin-1 from a cytosolic brain extract than capsids with more outer tegument [11]. Furthermore these outer tegument proteins might lead to an association with cytoplasmic membranes, although our quantitative electron microscopy data indicate that while HSV1-ΔUL20 was impaired in completing secondary envelopment, there was no higher association with wrapping membranes when compared to the respective parental strains. Those capsids associated with wrapping membranes might have been connected to larger membrane systems in neurons, which might have reduced their motility, and thus their chances to find the axonal exit. Axonal transport in general had not been impaired upon infection with either HSV-1 mutant, since the envelope proteins gB and gD as well as the outer tegument proteins VP13/14 and VP22 had been targeted to axons. Our data are consistent with the notion that cytosolic capsids might rely on the inner tegument for kinesin-1 mediated transport in epithelial cells and the somata, but that transport vesicles harboring complete virions are more efficiently targeted to axons than such cytosolic capsids.

### Married versus separate assembly models

Our data indicate that in addition to pUL36 and pUL37, other HSV-1 components and processes depending on pUL20 are required for axonal targeting in adult neurons. The simplest interpretation of our data is that HSV-1 relies also on the *married model* for axonal targeting and trafficking, and that the membrane of the transport vesicles harboring complete virions harbors a viral or a host receptor for *smart* axonal microtubule motors on its cytosolic surface. Indeed, kinesin-1 can co-traffic with PRV and HSV-1 tegument and envelope proteins [102–104]. In the absence of pUL20, secondary envelopment was not completed, and apparently also the tegument adopted a different conformation or composition, as our electron microscopy analysis revealed a different tegument contrast for capsids associated with wrapping membranes than for complete virions. Furthermore without pUL20, the envelope proteins gD and gH/gL, and possibly also their interaction partners VP22 and VP16 [38], are not properly targeted to cytoplasmic virus assembly sites [105].

Of the alphaherpesvirus envelope proteins, gE/gI and pUS9 are also necessary for efficient axonal spread but not for cytoplasmic capsid envelopment [23]. pUS9 of PRV associates with the neuron-specific kinesin-3 subunit KIF1A, and contributes to initial axonal sorting of PRV particles [106–108]. Similarly, pUS9 of HSV-1 is required for efficient axonal targeting, and its large cytosolic domain interacts with the KIF5B subunit of conventional kinesin-1 [74,109]. If the intracellular trafficking and targeting of gE/gI or pUS9 had been impaired in the absence of pUL20, this would explain why even capsids associated with wrapping membranes could not be targeted to the axons upon infection with HSV1-ΔUL20. According to the loading hypothesis, capsids require in the somata in addition to the inner tegument proteins pUL36 and pUL37 also the membrane proteins gE/gI and pUS9 to be loaded onto kinesin-1 [13,109,110].

Vesicles harboring gD and possibly VP22 did neither require pUL36, pUL37 nor pUL20 for axonal targeting. Future work elucidating which assembly intermediates of wild-type HSV-1 as well as -ΔUL36, -ΔUL37, -ΔUL20 and other deletion mutants recruit which *smart* microtubule motors will reveal specific viral and specific host components that need to be assembled onto an alphaherpesvirus cargo for efficient axonal targeting. Our data are consistent with the notion that cytosolic capsids rely on the inner tegument for dynein and kinesin-1 mediated transport prior to secondary envelopment in epithelial cells and in the neuronal somata, but that transport vesicles harboring complete virions are more efficiently targeted to axons than such cytosolic capsids.

## Materials and methods

### Cell lines

Cell lines were cultured in a humidified incubator at 37°C and 5% CO_2_ and passaged twice per week. BHK-21 cells (ATCC CCL-10) were maintained in MEM (Cytogen, Wetzlar, Germany) supplemented with 10% (v/v) FBS (fetal bovine serum; PAA Laboratories GmbH, Cölbe, Germany), Vero cells (ATCC CCL-81) in MEM supplemented with 7.5% (v/v) FBS, and HEK-293T cells (ATCC CRL-11268) and Flp-In-CV-1 cells in DMEM (Invitrogen, Karlsruhe, Germany) supplemented with 2 mM L-glutamine and 10% FBS (v/v). The pUL36 *trans*-complementing Vero-derived HS30 cell line [31] was provided by Prashant Desai (Johns Hopkins University, Baltimore, USA) and the pUL37 *trans*-complementing rabbit skin 80C02 cell line [15] by Frazer J. Rixon (University of Glasgow, Scotland, UK). These complementing cells were maintained in MEM containing 1% non-essential amino acids (Cytogen) and 7.5% or 10% (v/v) FBS, respectively. Every 5^th^ passage was cultured in the presence of G418 (500 μg/ml; PAA Laboratories GmbH). The *trans*-complementing Flp-In-CV-1-derived cell line expressing pUL20 under the control of the HSV-1 gD promoter [54] was provided by Konstantin G. Kousoulas (Louisiana State University, Louisiana, USA) and maintained in DMEM (Invitrogen) supplemented with 10% (v/v) FBS and 2 mM L-glutamine and 125 μg/ml hygromycin B (Invitrogen).

### Neurons

Primary neurons from dorsal root ganglia (DRG) of adult C57Bl/6JHanZtm mice were cultured using established protocols [111,112]. Briefly, mice were sacrificed, and the DRG from the cervical, thoracic and lumbar level were dissected and collected in 1x HBSS-complete buffer (Hank’s balanced salt solution, pH 7.4 with 5 mM HEPES and 10 mM D-Glucose). The DRG of three mice were pooled and treated with 20 mg/ml papain (Sigma-Aldrich, Schnelldorf, Germany; in 0.4 mg/ml L-Cysteine, 0.5 mM EDTA, 1.5 mM CaCl_2_, pH 7.4) for 20 min at 37°C, and with 10 mg/ml collagenase IV (Invitrogen) and 12 mg/ml dispase II (Sigma-Aldrich) in 1x HBSS-complete buffer for 20 min at 37°C. DRG and cells were sedimented and re-suspended in 1 ml 1xHBSS-complete buffer and triturated using Pasteur pipettes with narrowed ends. The suspensions were spun for 8 min at 381 x g through 20% (v/v) Percoll (Sigma-Aldrich) cushions in CO_2_-independent medium (Life Technologies Gibco, Carlsbad, CA, USA) containing 10 mM D-glucose, 5 mM HEPES, 10% FBS, 100 U/ml penicillin and 0.1 mg/ml streptomycin. The cells were washed with 2 ml CO_2_-independent medium, sedimented 2 min at 1,000 x g, suspended in Ham’s F-12 nutrient mix medium with 10% FBS, 50 ng/ml 2.5S nerve growth factor (Promega Corporation, Fitchburg, WI, USA), 100 U/ml penicillin and 0.1 mg/ml streptomycin, and seeded onto cover slips of 20 mm diameter in 24-well plates or glass bottom dishes (Nunc LabTek II Chambered cover glass 4-chamber #155382, #1.5 borosilicate glass, Thermo Scientific). The cover slips and glass bottom dishes had been pre-coated with 0.01% (w/v) poly-L-lysine (150,000–300,000 g/mol, Sigma-Aldrich) and 7 ng/μl murine laminin (Invitrogen). The cells were cultured in a humidified incubator at 37°C and 5% CO_2_, and the media were replaced twice a week. 1-β-D-arabinofuranosylcytosine (Sigma-Aldrich) was added at 1 to 2 div to a final concentration of 2μM to suppress proliferation of dividing, non-neuronal cells, but removed prior to HSV-1 infection.

### Antibodies

We used rabbit polyclonal antibodies (pAb) to detect VP26 [VP26aa95-112; 113], pUL36 [#147; 43], pUL37 [32], pUL20 [114], VP13/14 [R22, 115], gB [R69, 116] or VP16 (BD 3844–1, Becton-Dickinson, Franklin Lakes, NJ, USA). Mock infected neurons showed little binding to antisera raised against HSV-1 proteins that had been cleared by pre-adsorption [89] on uninfected neurons. Mouse monoclonal antibodies (mAb) were used to detect VP22 [mAb22-3; 117] or gD [mAb DL6; 118]. To detect host antigens, we used mAb 1501 for actin (Millipore, Billerica, MA, USA), mAb 5564 for β-III-tubulin (Millipore), mAb 106/36 for ankyrinG (E9PE32; UC Davis/NIH NeuroMab Facility), SMI-310 for phosphorylated 200 kDa and 160 kDa neurofilament (ab24570, Abcam, Cambridge, UK), and SMI-320 for non-phosphorylated 200 kDa neurofilament (ab28029, Abcam).

### Viruses

We used the clinical isolate HSV1(17^+^) [119] and its derivatives HSV1(17^+^)Lox [20], HSV1(17^+^)Lox-CheVP26 [20], HSV1(17^+^)Lox-ΔUL36 [20,47], HSV1(17^+^)Lox-CheVP26-ΔUL36 [20,47], HSV1(17^+^)Lox-ΔUL37 [20], HSV1(17^+^)Lox-CheVP26-ΔUL37 [20], HSV1(17^+^)Lox-ΔUL20 (see below) and HSV1(17^+^)Lox-CheVP26-ΔUL20 (see below). Our pHSV1(17^+^)Lox BAC plasmids contain a BAC cassette with a chloramphenicol resistance gene, a Cre recombinase gene with an intron under the control of a eukaryotic promoter, a single flippase recognition target site (FRT) and LoxP sites at both ends inserted between the genes UL22 and UL23, and an almost complete HSV1(17^+^) genome lacking only the Ori_L_ [84,85]. The Cre recombinase excises the BAC cassette upon transfection into eukaryotic cells. HSV-1 stocks were prepared in BHK-21, HS30 (for -ΔUL36), 80C02 (for -ΔUL37) or Flp-In-CV-1-pUL20 (for -ΔUL20), and extracellular virus was harvested by sedimentation from the supernatant of infected cells as described previously [84,89,90]. After DNase treatment, the inocula of HSV1(17^+^)Lox-ΔUL37 and Lox-CheVP26-ΔUL37 had genome to PFU ratios below 2,300 while that of the other strains had genome to PFU ratios of below 108 [120].

### Lentiviral production and transduction

To express EB3 in neurons, we used lentiviral transduction. The spleen focus forming virus promoter was replaced by the human cytomegalovirus immediate early promoter in the plasmid pRRL.PPT.SF.GFPpre [121, provided by Axel Schambach, Hannover Medical School, Hannover, Germany] via PCR by generating 5’*PstI* and 3’*BamHI* restriction sites adjacent to the HCMV immediate early promoter of pCMV-Tag 2B (Agilent Technologies, Santa Clara, California, USA) using the primers 5’-GAACCTGCAGCGTATTACCGCCATGCATTAGT-3’ and 5’-GAACGGATCCCCAGCTTTTGTTCCCTTTAGTG-3’. Furthermore, with the restriction sites 5’*NdeI* and 3’*AgeI*, parts of the human cytomegalovirus immediate early promoter and EB3 were cloned from pEGFP-N-EB3 [51, provided by Marco van Ham, Helmholtz Centre for Infection Research, Braunschweig, Germany] to generate pRRL.PPT.HCMV.GFPEB3pre. HEK 293T cells (5 x 10^6^ per 10 cm dish) were transfected with 5 μg pRSV_Rev (provided by Axel Schambach), 2 μg pMD2.g (Addgene Inc., Cambridge, MA, USA, Cat. No. 12259, deposited by D. Trono, provided by Axel Schambach), 10 μg pCDNA3.GP.CCCC (provided by Axel Schambach), and 10 μg transfer plasmid as described previously [122]. The supernatants were harvested and spun in a Beckman SW 28 rotor at 23,000 rpm or a SW32.Ti rotor at 24,000 rpm for 90 min at 4°C (Beckman Coulter, Krefeld, Germany). The resuspended lentiviral particles were snap frozen in liquid N_2_ and stored at -80°C. For lentiviral transduction, DRG neurons were prepared and seeded as described and at 1 div neuronal growth medium was replaced by one containing 20 mM HEPES and the lentiviral particles.

### Plaque assay

HSV-1 inocula titers were determined by plaque assays [89,90,120]. Vero, Flp-In-CV-1 or the derivative Flp-In-CV-1-UL20-expressing cells were cultured in 6-well dishes for 16 to 20 h to almost confluency. The respective inocula were diluted in CO_2_-independent medium (Life Technologies Gibco) containing 0.1% (w/v) cell-culture grade bovine serum albumin (BSA; Capricorn Scientific, Ebersdorfergrund, Germany), added to the cells for 1 h on a rocking platform at room temperature, and then replaced by growth medium containing 20 μg/ml pooled human IgGs (Sigma-Aldrich) to neutralize HSV-1 in the culture medium. At 3 dpi, the cells were fixed with ice-cold, water-free methanol and air-dried prior to staining with 0.1% (w/v) crystal violet in 2% (v/v) ethanol for 1 min. After removing the excess of crystal violet, the cells were air-dried, and plaques were counted while using a binocular loupe (Nikon, Tokyo, Japan) to calculate the virus titer as plaque forming units (pfu) per ml.

### HSV-1 BAC mutagenesis and one-step growth curve

We used the BACs pHSV1(17^+^)Lox and pHSV1(17^+^)Lox-CheVP26 to construct HSV1(17^+^)Lox-ΔUL20 and HSV1(17^+^)Lox-CheVP26-ΔUL20. To prevent expression of pUL20, we mutated the ATG-start codon and a second ATG codon of UL20 to CTG followed by an immediate insertion of 3 stop codons. Recombinant PCR fragments of pEPkan-S2 [provided by B. Karsten Tischer and Nikolaus Osterrieder, Freie Universität Berlin, Germany, 123] were amplified to mutate the 5’ region of UL20 using traceless Red recombination, and transformed for homologous recombination into E. coli GS1783 (provided by G. Smith, Northwestern University, Chicago, IL, USA) harboring pHSV1(17^+^)Lox or pHSV1(17^+^)Lox-CheVP26 [85,124]. We used the forward primer 5’-CCTTGCGGTTTCGGTCTCCCCACCTCCACCGCACACCCCCTGACCCTG**TAGTAATAG**CGGGATGACCTTCCTCTGGTTAGGGAAACAGGTAATCGATTT-3’, the reverse primer 5’-TCGTCGACCAGATCTCGATCACCAGAGGAAGGTCATCCCG**CTATTACTA**CAGGGTCAGGGGGTGTGCGGTGGCAGGTGGTGCCAGTGTTACAACCAATTAACC-3’, the forward sequencing primer 5’-AAAGACCGGCTGGGTATG-3’, and the reverse sequencing primer 5’-GGGCGTAGGCGTAAATTC-3’. The mutated start codons are underlined and the inserted stop codons are shown in bold. The BAC plasmids were digested with 15 U/μg DNA of *AscI*, *BamHI*, *XhoI* or *HindIII* for 3.5 h, and analyzed on 0.6% (w/v) agarose gels in 0.5x TBE buffer (0.44 M Tris-HCl, 0.44 mM boric acid, 10 mM EDTA, pH 8) run at 66 mA for 17 h (Peqlab system, Erlangen, Germany). To reconstitute viruses, sub-confluent Vero or Flp-In-CV-1-UL20-expressing cells were transfected with 10 μg BAC-DNA (MBS mammalian transfection kit; Stratagene, La Jolla, CA, USA) per 6 cm dish and cultured until cytopathic effects had developed. Cells and medium were pooled, virus was released by three cycles of freeze-thawing and the resulting cell pellet was used to further propagate the virus according to standard protocols. The genomes of the novel HSV1(17^+^)Lox strains were sequenced around the pUL20 start site that had been targeted, and the engineered mutations were confirmed. For the growth curves, sub-confluent Vero, Flp-In-CV-1 or the derivative Flp-In-CV-1-UL20-expressing cells were inoculated at 5 pfu/cell, the cells and the supernatants were harvested at the indicated time points, and virus titers were determined by plaque assay.

### HSV-1 infection

Vero cells were seeded on cover slips, 3.5 cm cell culture dishes or glass-bottom chambers (Nunc LabTek II Chambered cover glass 4-chamber #155382, #1.5 borosilicate glass, Thermo Scientific) and infected 16 to 20 hours after the seeding as described before [16,20,29,84,89,120,125]. For synchronous infections, the cells were pre-cooled for 20 min on ice, and inoculated with 10 pfu per cell or mock treated as a control in CO_2_-independent medium containing 0.1% (w/v) BSA for 2 h on ice while rocking. The cells were then shifted to regular growth medium at 37°C and 5% CO_2_ for 1 h. Non-internalized virus was inactivated at 4°C by a 3 min acid wash (40 mM citrate, 135 mM NaCl, 10 mM KCl, pH 3). Neurons cultured on cover slips in 24-well plates were pre-incubated at room temperature with CO_2_-independent medium for 20 min, and inoculated with 1 to 5 × 10^6^ PFU in 200 μl per well in CO_2_-independent medium. After 30 min, the virus-suspension was replaced by 500 μl F-12 medium and cells were incubated again at 37°C and 5% CO_2_.

### Immunoblot

Cells were lysed with hot sample buffer (50 mM Tris-HCl, pH 6.8, 1% [w/v] SDS, 1% [v/v] β-mercaptoethanol, 5% [v/v] glycerol, 0.001% [w/v] bromophenol blue) containing protease inhibitors AEL (aprotinin, E-64, leupeptin, Sigma), ABP (antipain, bestatin, pepstatin, Sigma) and PMSF (Roth, Karlsruhe, Germany). The proteins were separated by SDS-PAGE in 12.5% gels and transferred in 48 mM Tris, 380 mM glycine, 0.1% [w/v] SDS and 10% [v/v] methanol to nitrocellulose membranes (Pall Corporation, Pensacola, FL, USA). After blocking with 5% (w/v) low-fat milk powder in PBS containing 0.1% (v/v) Tween 20, the membranes were incubated with primary antibodies and secondary antibodies coupled to alkaline phosphatase (Dianova, Hamburg, Germany), transferred to 100 mM Tris-HCl, pH 9.5, 100 mM NaCl, 5 mM MgCl_2_, and stained with 0.2 mM nitroblue tetrazolium chloride and 0.8 mM 5-bromo-4-chloro-indolyl-3-phosphate. For documentation, the membranes were imaged with a digital scanner (ScanJet 6300, Hewlett Packard, Wilmington, DE, USA).

### Fluorescence microscopy

Cells were fixed at room temperature with 3% (w/v) paraformaldehyde (PFA) in PBS for 20 min, followed by 50 mM NH_4_Cl for 10 min and permeabilization with 0.1% Triton-X-100 for 5 min, or at 37°C with PHEMO fix [3.7% (w/v) PFA, 0.05% [w/v] glutaraldehyde, 0.5% [v/v] Triton-X-100 in PHEMO buffer with 68 mM PIPES, 25 mM HEPES, pH 6.9, 15 mM EGTA, 3 mM MgCl_2_, 10% [v/v] dimethyl sulfoxide], washed two times with PHEMO buffer followed by the NH_4_Cl treatment. The HSV-1 Fc-receptor and unspecific protein binding sites were blocked with 10% (v/v) human serum of HSV-1-seronegative healthy volunteers and 0.5% (w/v) BSA. Samples were labeled with primary and pre-adsorbed secondary antibodies to prevent cross reactivity to antibodies of other species, namely goat-anti-mouse coupled with rhodamine X, carbocyanine 5 or AlexaFluor488, and goat-anti-rabbit coupled with AlexaFluor488 (Invitrogen). DNA was stained using To-Pro-3-iodide or DAPI dyes (Invitrogen), and the cover slips were mounted in Mowiol 4–88 containing 10% (w/v) 1,4-diazabicyclo-[2.2.2]octane. The specimens were analyzed by confocal fluorescence microscopy (LSM 510 Meta, software LSM 510 version 4, ZEISS, Göttingen, Germany). Pseudo-coloring, brightness and contrast were adjusted identically across each set of images using Adobe Photoshop CS4.

### Automated image processing

To analyze systematically the degree of co-localization of tegument (anti-VP22, red channel) or envelope proteins (anti-gD, red channel) with capsids (anti-VP26, green channel), and the targeting efficiency of the different viral particles to the axons, we developed a novel image processing pipeline. The semi-automated approach consists of three steps: (i) manual annotation of regions of interest (ROIs) to identify the axons (cf. S4 Fig), (ii) automated particle detection of the capsids, and (iii) automated detection of the co-localizing tegument or envelope protein signal using pixel-wise image classification.

*(i) ROI selection*: To distinguish the axon from other structures, the regions of interest were manually selected. To simplify the process of region annotation, a ROI is defined by positioning a few control points in the image and adjusting the region width at the control points, as shown in S4 Fig. Usually, three to four control points were sufficient for accurate annotation.

*(ii) Capsid particle detection*: The capsids have a spherical shape with a characteristic diameter. Due to blurring caused by the image acquisition process, the fluorescence signal of a single capsid was modeled by an isotropic Gaussian distribution. Template matching was applied to discriminate between well-separated viral particles and other signals such as background noise or agglomerations of multiple, non-separable capsids. In detail, a detector response map R_σ_(x, y) was computed for each pixel (x, y) of the image using normalized cross-correlation of the image signal *I*_capsid_(*x*,y) with a Gaussian shaped template model $T_{\sigma }(u,v)=exp[-\frac{u^{2}+v^{2}}{2\sigma^{2}}]$ with variance σ^2^ and filter size of 1 + 2 ⋅ ⌈*σ* ⋅ *s*⌉. Candidate particles were extracted from the detector response map R_σ_(x, y) by searching for local maxima in R_σ_(x, y) using an 8-neighborhood. In case of a plateau, a morphological shrinking operator was used to only consider the central position of the plateau as a detection candidate. To discriminate from noise, the candidate points p_i_ = (x_i_, y_i_) were only accepted as capsids if the detector response *R*_*σ*_(*x*_*i*_,*y*_*i*_) was greater than a threshold *τ*_corr_ and the fluorescence signal *I*_capsid_(*x*_*i*_,*y*_*i*_) was greater than a threshold *τ*_intens_. The parameters *σ*, *s*, *τ*_corr_, and *τ*_intens_ were trained on manually annotated test images using grid search. The final parameter values used throughout the experiments were *σ* = 0.7, *s* = 1.5, *τ*_corr_ = 0.4, and *τ*_intens_ = 0.5.

*(iii) Tegument or envelope protein signal detection*: We used pixel-wise image classification in order to discriminate between signal and background noise. The classifier used various features derived from the gD or VP22 fluorescence signals *I*_gD_(x,y): Gaussian blurred intensity signal $I_{\sigma }^{gD}=I_{gD}*T_{\sigma }$, non-linearly distorted intensity $E_{s;\sigma }^{gD}=exp(s\cdot I_{\sigma }^{gD})$ normalized cross correlation (NCC) of *I*_gD_ to a Gaussian kernel $R_{\sigma }^{gD}=NCC(I_{gD},T_{\sigma })$, and a high-pass filter clamped to values between 0 and 1. Finally, the feature vector is

$$F=[R_{0.5}^{gD},R_{1}^{gD},R_{2}^{gD},R_{4}^{gD},I_{gD},E_{10;0}^{gD},I_{2}^{gD},H_{2}^{gD},I_{5}^{gD},H_{5}^{gD},E_{10;5}^{gD},I_{10}^{gD},H_{10}^{gD},E_{20;10}^{gD},I_{20}^{gD},H_{20}^{gD},E_{50;20}^{gD}].$$

We used a logistic regression classifier which was trained on manually annotated test images. The gD or VP22 regions were extracted from the resulting pixel-wise classification images using a connected components analysis with an 8-neighborhood. A capsid was said to co-localize with gD or VP22 if the capsid center position coincided with a gD or VP22 region dilated by 1 pixel. The dilation operation accounted for uncertainties in both the capsid position estimation and the pixel-wise gD or VP22 classification.

Several statistical parameter were derived from the automatically detected capsids and gD or VP22 regions. The number of capsids or gD and VP22 regions per 100 μm was derived by projecting the particle locations orthogonally to the axial center line of the ROI as illustrated in S4 Fig.

### Live cell imaging and particle tracking

For differential interference contrast imaging and to avoid evaporation at 37°C, a glass lid was attached to the chambers with vacuum grease (Dow Corning, Midland, Michigan, USA). Movies of infected Vero cells were recorded at 37° with a high temporal resolution of 5 images per second and a pixel size of 79 nm using a confocal laser scanning microscope equipped with a heating unit (PeCon, Erbach, Germany) as described before [20]. Movies of infected DRG neurons were recorded at 37°C (Incubator from PeCon, Erbach, Germany) with high temporal resolution 20 images per second on a Nikon Ti microscope equipped with a Yokogawa (Ratingen, Germany) CSU-X1 spinning disk and an Andor iXion Ultra 897 EMCCD camera (Belfast, UK).

### Digital movie processing

Automated tracking of cytoplasmic capsid motility was performed using the ImageJ plugin MOSAIC [63,126]. First, the nuclei were identified by the confined nuclear capsids mobility and the nuclear capsid fluorescence was removed. Second, the Gaussian blur filter of ImageJ was applied (Sigma radius 2 pixel). No further image processing was performed. Third, automated tracking was performed using the Particle Tracking plugin with the following settings: Kernel radius 3, Cutoff radius 3, percentile 0.5, displacement 20, and link range 1. Of the tracks identified, we only considered further tracks with a length of 10 frames or longer. Track length and maximum step velocity were calculated from tracks with a MSD exponent of 1.2 or larger, representing non diffusive transport events.

### Electron microscopy

Cells were infected as described above and fixed at the indicated time points. For conventional electron microscopy cells were fixed with 2% glutaraldehyde in cacodylate buffer (130 mM (CH_3_)_2_AsO_2_H, pH 7.4, 2 mM CaCl_2_, 10 mM MgCl_2_) for 1 h at room temperature. Cells were washed and contrasted with 1% (w/v) OsO_4_ in cacodylate buffer (165 mM (CH_3_)_2_AsO_2_H, pH 7.4, 1.5% (w/v) K_3_[Fe(CH)_6_] followed by 0.5% (w/v) uranyl acetate in 50% (v/v) ethanol overnight. The cells were embedded in Epon plastic (Serva, Heidelberg, Germany) and 50 nm ultrathin sections were cut parallel to the substrate. Cytoplasmic capsids were counted and the respective cellular areas that had been sampled were measured using an Image J plugin.

For immunoelectron microscopy, infected cells were fixed at the indicated time points with 2% (w/v) PFA and 0.2% [w/v] glutaraldehyde, in PHEM-buffer (60 mM PIPES, 25 mM HEPES, pH 6.9, 10 mM EGTA, 2 mM MgCl_2_), embedded, frozen and sectioned. Sections were labeled using specific antibodies and protein-A gold of 10 nm (Cell Microscopy Centre, Utrecht School of Medicine, The Netherlands). Sections were contrasted using 0.5% (w/v) uranyl acetate in 2% methylcellulose (Merck) [127]. Images were acquired with an electron microscope at 200 kV equipped with an Eagle 4k camera (Tecnai G2; FEI, Eindhoven, The Netherlands). Immunogold labeling was quantified by counting each gold particle within a radius of 100 nm around the center of a cytoplasmic capsid [20]. Data were analyzed by using Kruskal-Wallis followed by Dunn’s post testing, and the p values were adjusted for multiple testing (software Prism, version 6; Graphpad, San Diego, CA, USA).

### Ethic statement

The mice (strain C57Bl/6JHanZtm, not genetically modified) were bred and maintained without any perturbation. On the day of the experiment they were picked up from the animal facility and within 3 h sedated with CO_2_-inhalation prior to killing by cervical dislocation without any prior experimental perturbation. DRG were dissected afterwards. According to the German Animal Welfare Law §4, killing of animals does not need approval if the removal of organs serves scientific purposes and the mice had not undergone experimental treatment before. The animal care and sacrifice was performed in strict accordance with the German regulations of the Society for Laboratory Animal Science (GV-SOLAS), the European Health Law of the Federation of Laboratory Animal Science Association (FELASA) and the German Animal Welfare Law. According to the German Animal Welfare Law, this study does not contain animal experiments that require pre-approval, but the total number of killed mice was reported at the end of each year to the animal welfare deputy of Hannover Medical School. The number of animals killed according to §4 of the German Animal Welfare Law was registered with the animal welfare application number 2012/20 at the LAVES (*Niedersaechsisches Landesamt fuer Verbraucherschutz und Lebensmittelsicherheit*, *Oldenburg*, Germany), and the experiments were performed before 2013. Human sera of adult, healthy, HSV-1 seronegative volunteers were obtained after written informed consent by the blood donors. Permission was granted by the Institution Review Board (Hannover Medical School; Approval Number 893).

## Supporting information

S1 Fig Neurons from dorsal root ganglia of adult mice have polarized axons. DRG neurons were cultured for 5 div, fixed using the PHEMO (A-C) or the PFA/TX-100 protocol (D) and labeled with antibodies against β-III-tubulin (A), phosphorylated epitopes in neurofilament H and M (B), non-phosphorylated neurofilament H (C), or ankyrinG (D). Differential interference contrast images (i) and fluorescence (ii). Aii is one confocal slice, Bii and Cii are projections of 3 confocal slices, Dii is a projection of 7 confocal slices. Scale bars, 10 μm.
(TIF)

S2 Fig Characterization of HSV1(17^+^)Lox-ΔUL20 and Lox-CheVP26-ΔUL20 mutants. (A) Purified HSV1 BAC DNA was digested with the restriction enzymes *XhoI*, *AscI*, *BamHI* or *HindIII*, and the fragments were analyzed on agarose gels. c, bands containing the mCherry-tag; star, bands containing the mutation. (B) Immunoblot analysis of infected cell lysates. Vero cells were infected with HSV1(17^+^)Lox or its derivatives -ΔUL20, -CheVP26, or CheVP26-ΔUL20 at an MOI of 10 pfu/cell (3.2 x 10^7^ pfu/mL), and lysed in sample buffer at the indicated time points. The proteins were separated with a 12.5% SDS-PAGE, transferred to a nitrocellulose membrane, and probed with antibodies directed against pUL37, pUL20, or actin. (C) and (D) Single step growth kinetics of parental and mutant strains, either black (squares) or tagged with CheVP26 (circles) on either Vero cells (filled and empty symbols) or on Flp-In™-CV-1-pUL20-complementing cells (half-filled symbols). Intracellular infectivity (C) and extracellular infectivity (D).
(TIF)

S3 Fig HSV-1-pUL36, pUL37 and pUL20 affect the subcellular capsid distribution in the cell body. DRG neurons were infected after 3 div with 1 x 10^7^ pfu/mL of HSV1(17^+^)Lox-CheVP26 (A), -ΔUL36 (B), -ΔUL37 (C), or -ΔUL20 (D), fixed and permeabilized using the PHEMO protocol at 24 hpi, labeled with antibodies directed against gB (R69, ii) or β-III-tubulin (mAb 5564, iii) and the cell bodies were analyzed by confocal microscopy. CheVP26 (i), merge (v), DIC (v). Scale bar is 5 μm.
(TIF)

S4 Fig Region of Interest annotation of the axons and computation of particles per μm. To quantify the amount of viral structures within axons, we collected random images of axonal regions. The schematic overview shows an example of a manual region of interest (ROI) annotation (green transparent tetragons) via control points (blue) and the computation of the axon length via orthogonal projection of the particle position (red point) on the axial centerline of the ROI (black line). The number of structures within such an ROI was determined using a novel automated image analysis algorithm.
(TIF)

S1 Movie Vero cells were infected with 6.4 x 10^6^ pfu/mL (10 pfu/cell) of HSV1(17^+^)Lox-CheVP26. Live cell imaging was performed between 8 and 10 hpi using fluorescence confocal microscopy and collecting five images per second for a total time period of 48 s. The Che channel was recorded and movie images were inverted. Individual capsids were automatically tracked. Tracks are indicated by a colored line; the front of each track marked by an open circle. The stills of the movie and the quantification of the tracks are shown in Fig 4.
(AVI)

S2 Movie Vero cells were infected with 6.4 x 10^6^ pfu/mL (10 pfu/cell) of HSV1(17^+^)Lox-CheVP26-ΔUL36. Live cell imaging was performed between 8 and 10 hpi using fluorescence confocal microscopy and collecting five images per second for a total time period of 48 s. The Che channel was recorded and movie images were inverted. Individual capsids were automatically tracked. Tracks are indicated by a colored line; the front of each track marked by an open circle. The stills of the movie and the quantification of the tracks are shown in Fig 4.
(AVI)

S3 Movie Vero cells were infected with 6.4 x 10^6^ pfu/mL (10 pfu/cell) of HSV1(17^+^)Lox-CheVP26-ΔUL37. Live cell imaging was performed between 8 and 10 hpi using fluorescence confocal microscopy and collecting five images per second for a total time period of 48 s. The Che channel was recorded and movie images were inverted. Individual capsids were automatically tracked. Tracks are indicated by a colored line; the front of each track marked by an open circle. The stills of the movie and the quantification of the tracks are shown in Fig 4.
(AVI)

S4 Movie Vero cells were infected with 6.4 x 10^6^ pfu/mL (10 pfu/cell) of HSV1(17^+^)Lox-CheVP26-ΔUL20. Live cell imaging was performed between 8 and 10 hpi using fluorescence confocal microscopy and collecting five images per second for a total time period of 48 s. The Che channel was recorded and movie images were inverted. Individual capsids were automatically tracked. Tracks are indicated by a colored line; the front of each track marked by an open circle. The stills of the movie and the quantification of the tracks are shown in Fig 4.
(AVI)

S5 Movie DRG neurons were infected with 1 x 10^7^ pfu/mL with HSV1(17^+^)Lox-CheVP26. Live cell imaging was performed between 22 and 31 hpi using spinning disk microscopy with 20 images per second. The Che channel was recorded and movie images were inverted. Individual capsids were automatically tracked. The tracks are indicated by a colored line. The stills of the movie and the quantification of the tracks are shown in Fig 5.
(AVI)

S6 Movie DRG neurons were infected with 1 x 10^7^ pfu/mL with HSV1(17^+^)Lox-CheVP26-ΔUL36. Live cell imaging was performed between 22 and 31 hpi using spinning disk microscopy with 20 images per second. The Che channel was recorded and movie images were inverted. Individual capsids were automatically tracked. The tracks are indicated by a colored line. The stills of the movie and the quantification of the tracks are shown in Fig 5.
(AVI)

S7 Movie DRG neurons were infected with 1 x 10^7^ pfu/mL with HSV1(17^+^)Lox-CheVP26-ΔUL37. Live cell imaging was performed between 22 and 31 hpi using spinning disk microscopy with 20 images per second. The Che channel was recorded and movie images were inverted. Individual capsids were automatically tracked. The tracks are indicated by a colored line. The stills of the movie and the quantification of the tracks are shown in Fig 5.
(AVI)

S8 Movie DRG neurons were infected with 1 x 10^7^ pfu/mL with HSV1(17^+^)Lox-CheVP26-ΔUL20. Live cell imaging was performed between 22 and 31 hpi using spinning disk microscopy with 20 images per second. The Che channel was recorded and movie images were inverted. Individual capsids were automatically tracked. The tracks are indicated by a colored line. The stills of the movie and the quantification of the tracks are shown in Fig 5.
(AVI)

S1 Table Quantification of HSV1 capsid axonal targeting details. (DOCX)
