# Supplementary figures and images for: Inner tegument proteins of Herpes Simplex Virus are sufficient for intracellular capsid motility in neurons but not for axonal targeting
Source: PLoS Pathog. 2017 Dec 28;13(12):e1006813. doi: 10.1371/journal.ppat.1006813 (PMC5761964; doi:10.1371/journal.ppat.1006813)

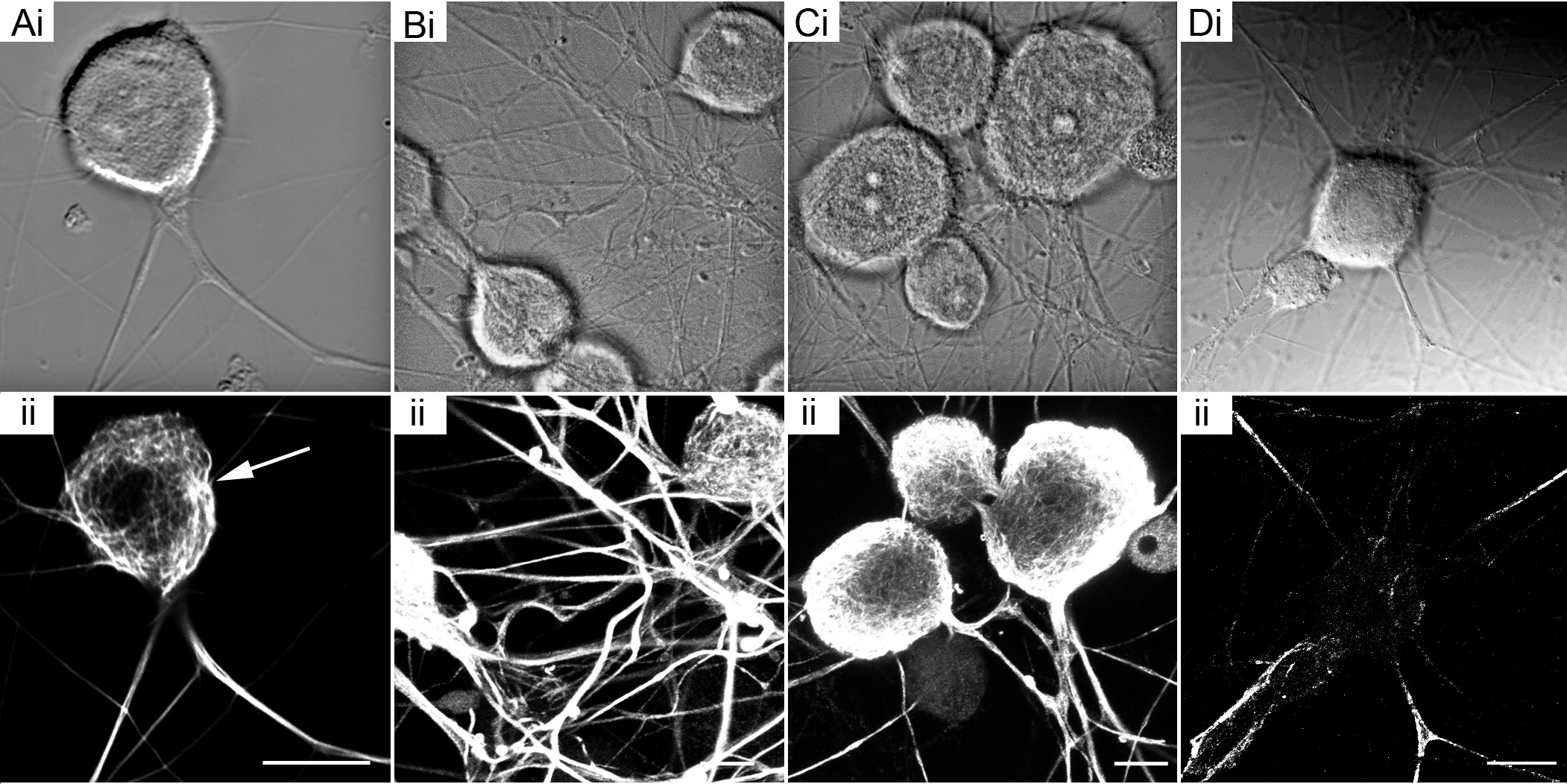

Supplement: S1 Fig — DRG neurons were cultured for 5 div, fixed using the PHEMO (A-C) or the PFA/TX-100 protocol (D) and labeled with antibodies against β-III-tubulin (A), phosphorylated epitopes in neurofilament H and M (B), non-phosphorylated neurofilament H (C), or ankyrinG (D). Differential interference contrast images (i) and fluorescence (ii). Aii is one confocal slice, Bii and Cii are projections of 3 confocal slices, Dii is a projection of 7 confocal slices. Scale bars, 10 μm. (TIF) [file ppat.1006813.s001.tif]

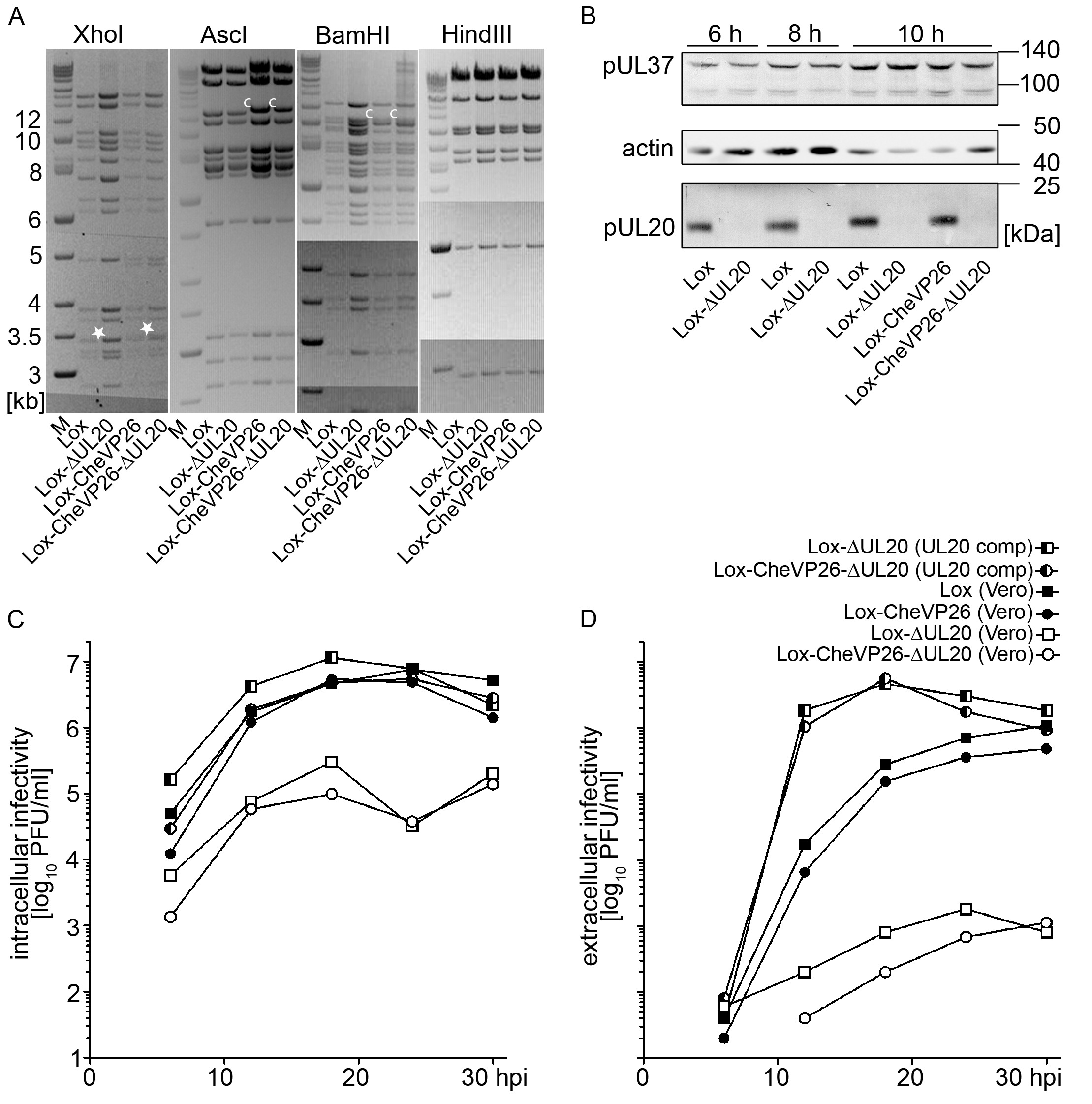

Supplement: S2 Fig — (A) Purified HSV1 BAC DNA was digested with the restriction enzymes XhoI, AscI, BamHI or HindIII, and the fragments were analyzed on agarose gels. c, bands containing the mCherry-tag; star, bands containing the mutation. (B) Immunoblot analysis of infected cell lysates. Vero cells were infected with HSV1(17+)Lox or its derivatives -ΔUL20, -CheVP26, or CheVP26-ΔUL20 at an MOI of 10 pfu/cell (3.2 x 10^7^ pfu/mL), and lysed in sample buffer at the indicated time points. The proteins were separated with a 12.5% SDS-PAGE, transferred to a nitrocellulose membrane, and probed with antibodies directed against pUL37, pUL20, or actin. (C) and (D) Single step growth kinetics of parental and mutant strains, either black (squares) or tagged with CheVP26 (circles) on either Vero cells (filled and empty symbols) or on Flp-In™-CV-1-pUL20-complementing cells (half-filled symbols). Intracellular infectivity (C) and extracellular infectivity (D). (TIF) [file ppat.1006813.s002.tif]

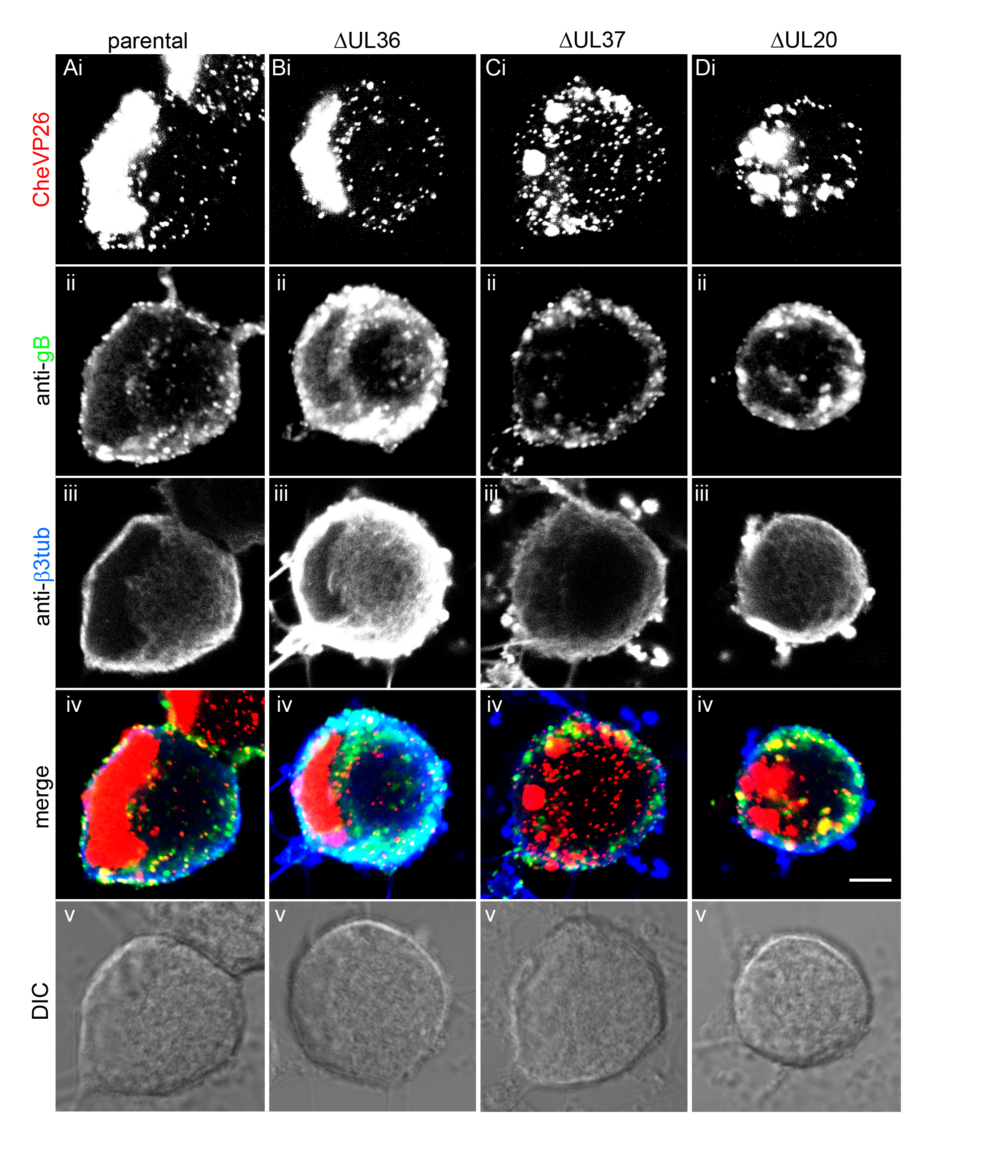

Supplement: S3 Fig — DRG neurons were infected after 3 div with 1 x 10^7^ pfu/mL of HSV1(17+)Lox-CheVP26 (A), -ΔUL36 (B), -ΔUL37 (C), or -ΔUL20 (D), fixed and permeabilized using the PHEMO protocol at 24 hpi, labeled with antibodies directed against gB (R69, ii) or β-III-tubulin (mAb 5564, iii) and the cell bodies were analyzed by confocal microscopy. CheVP26 (i), merge (v), DIC (v). Scale bar is 5 μm. (TIF) [file ppat.1006813.s003.tif]

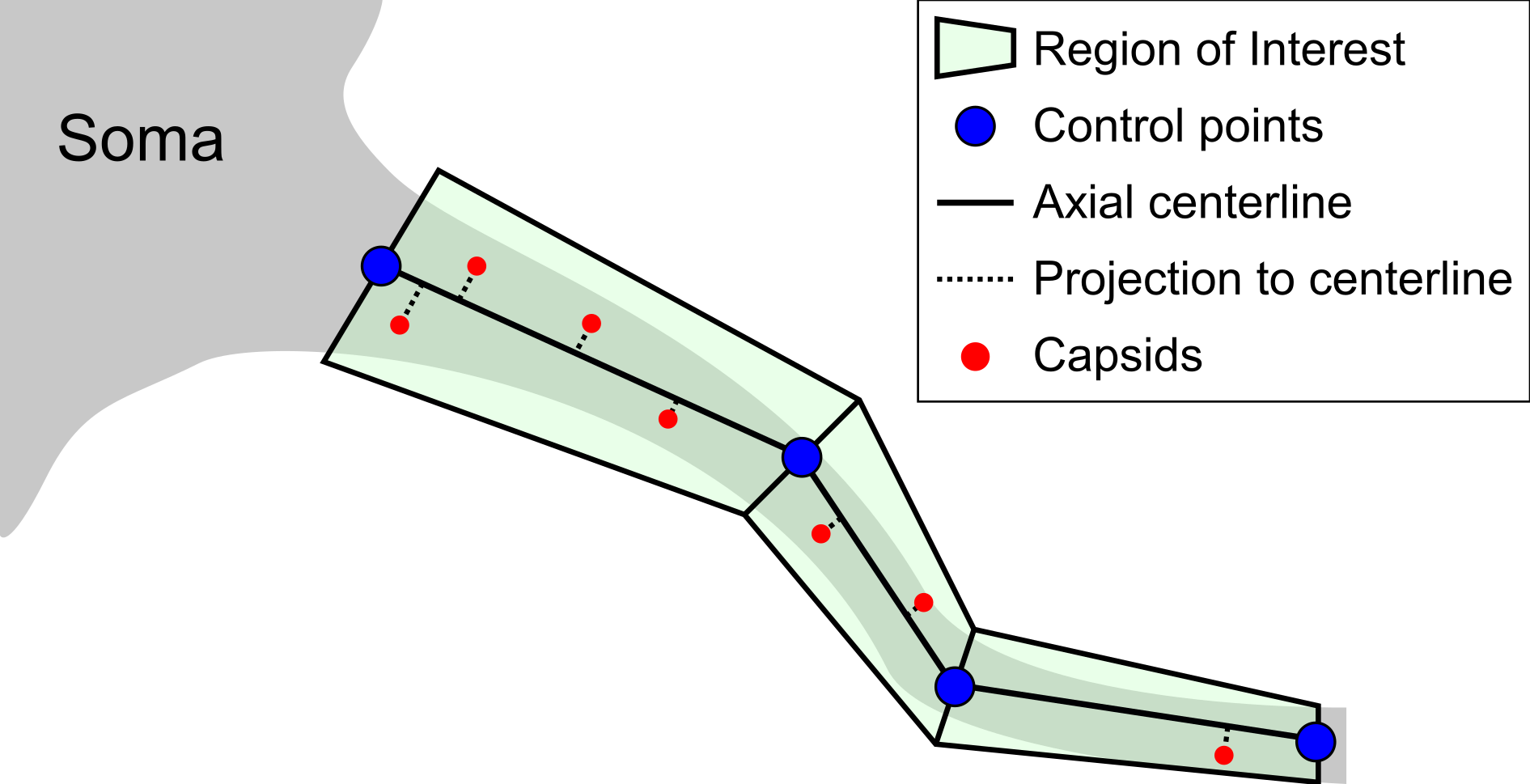

Supplement: S4 Fig — To quantify the amount of viral structures within axons, we collected random images of axonal regions. The schematic overview shows an example of a manual region of interest (ROI) annotation (green transparent tetragons) via control points (blue) and the computation of the axon length via orthogonal projection of the particle position (red point) on the axial centerline of the ROI (black line). The number of structures within such an ROI was determined using a novel automated image analysis algorithm. (TIF) [file ppat.1006813.s004.tif]
