# Supplementary material for: Inner tegument proteins of Herpes Simplex Virus are sufficient for intracellular capsid motility in neurons but not for axonal targeting
Source: PLoS Pathog. 2017 Dec 28;13(12):e1006813. doi: 10.1371/journal.ppat.1006813 (PMC5761964; doi:10.1371/journal.ppat.1006813)
Supplement: S1 Table — (DOCX) [file ppat.1006813.s013.docx]

Table S1: Quantification of HSV1 capsid axonal targeting details

| **Fig 8** | **HSV1(17^+^)Lox** | **Exp. #** | **mock** | **parental** | **ΔUL36** | **ΔUL20** |
| --- | --- | --- | --- | --- | --- | --- |
| **anti-VP26**  **+**  **anti-VP22** | # of images | 1 | 16 | 23 | 26 | 24 |
|  |  | 2 | 7 | 12 | 12 | 11 |
|  | # of capsids | 1 | 2 | 829 | 46 | 71 |
|  |  | 2 | 0 | 338 | 40 | 102 |
|  | # of gD-positive structures | 1 | 155 | 599 | 543 | 501 |
|  |  | 2 | 30 | 374 | 193 | 345 |
|  | axon length [µm] | 1 | 1232 | 2915 | 3048 | 3077 |
|  |  | 2 | 502 | 1415 | 1084 | 1439 |
| **anti-VP26**  **+**  **anti-VP22** | # of images | 1 | 18 | 18 | 30 | 25 |
|  |  | 2 | 11 | 6 | 11 | 11 |
|  | # of capsids | 1 | 3 | 428 | 80 | 126 |
|  |  | 2 | 0 | 302 | 145 | 64 |
|  | # of VP22-positive structures | 1 | 2 | 396 | 622 | 399 |
|  |  | 2 | 1 | 364 | 110 | 52 |
|  | axon length [µm] | 1 | 1170 | 1753 | 3382 | 2725 |
|  |  | 2 | 543 | 1022 | 1137 | 1102 |
